# Dectin-1 Controls TSLP-Induced Th2 Response by Regulating STAT3, STAT6, and p50-RelB Activities in Dendritic Cells

**DOI:** 10.3389/fimmu.2021.678036

**Published:** 2021-07-07

**Authors:** Chao Gu, Katherine Upchurch, Joshua Horton, Mathew Wiest, Sandra Zurawski, Mark Millard, Robert R. Kane, HyeMee Joo, Lisa A. Miller, SangKon Oh

**Affiliations:** ^1^ Department of Immunology, Mayo Clinic, Scottsdale, AZ, United States; ^2^ Institute of Biomedical Studies, Baylor University, Waco, TX, United States; ^3^ Baylor Research Institute, Dallas, TX, United States; ^4^ Department of Pulmonology, Baylor University Medical Center, Dallas, TX, United States; ^5^ Department of Chemistry and Biochemistry, Baylor University, Waco, TX, United States; ^6^ California National Primate Research Center, University of California, Davis, Davis, CA, United States

**Keywords:** Dectin-1, TSLP, OX40L, dendritic cells, STAT3, STAT6, Th2 cells, allergy

## Abstract

The epithelium-associated cytokine thymic stromal lymphopoietin (TSLP) can induce OX40L and CCL17 expression by myeloid dendritic cells (mDCs), which contributes to aberrant Th2-type immune responses. Herein, we report that such TSLP-induced Th2-type immune response can be effectively controlled by Dectin-1, a C-type lectin receptor expressed by mDCs. Dectin-1 stimulation induced STAT3 activation and decreased the transcriptional activity of p50-RelB, both of which resulted in reduced OX40L expression on TSLP-activated mDCs. Dectin-1 stimulation also suppressed TSLP-induced STAT6 activation, resulting in decreased expression of the Th2 chemoattractant CCL17. We further demonstrated that Dectin-1 activation was capable of suppressing ragweed allergen (Amb a 1)-specific Th2-type T cell response in allergy patients *ex vivo* and house dust mite allergen (Der p 1)-specific IgE response in non-human primates *in vivo*. Collectively, this study provides a molecular explanation of Dectin-1-mediated suppression of Th2-type inflammatory responses and suggests Dectin-1 as a target for controlling Th2-type inflammation.

**Graphical Abstract:**
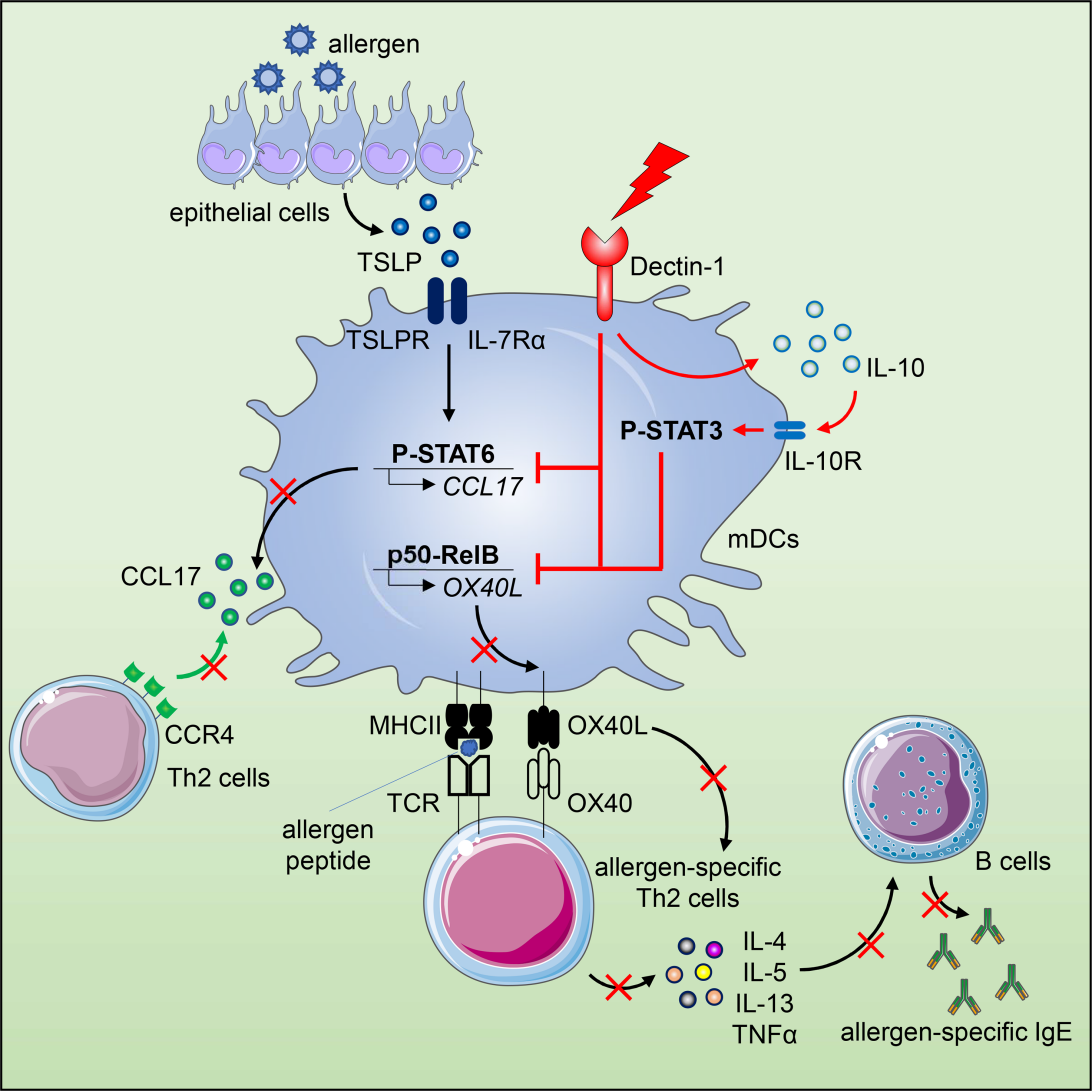


## Introduction

Human thymic stromal lymphopoietin (TSLP), an IL-7-like cytokine, is mainly produced by epithelial cells upon allergen exposure, with the highest expression in the lung, skin and gut (1, 2). A direct link between TSLP and pathogenesis of allergic diseases including atopic dermatitis, allergic asthma and allergic rhinitis has been reported (1, 3, 4). A growing body of evidence further suggests that TSLP acts as a master switch for allergic inflammation through licensing DCs to initiate inflammatory Th2 response (5, 6). Indeed, TSLP instructs myeloid DCs (mDCs) to prime Th2 cells in a unique two-part way. First, TSLP induces mDC maturation without driving cytokine production including IL-1α, IL-1β, IL-4, IL-6, IL-12, IL-23, IL-27, IFNα, IFNβ, IFNγ, TNFα and IL-10, thus creating a Th2-permissive microenvironment (1, 5). Second, TSLP induces surface OX40L expression by mDCs, which serves as the positive Th2-polarizing signal to directly trigger the differentiation of Th2 cells from naïve CD4^+^ T cells (5). More importantly, OX40L expressed by TSLP-activated mDCs (referred to hereafter as TSLP-mDCs) inhibits IL-10 production and promotes TNFα expression by Th2 cells, which endows Th2 cells with a TNFα^+^IL-10^-^ inflammatory phenotype (5). In addition to priming inflammatory Th2 response, TSLP-mDCs also play an important role in maintaining and further polarizing Th2 memory cells in allergic disease *via* an OX40L-dependent manner (7). We and others further showed that the interaction between OX40L and its cognate receptor, OX40, expressed on T cells can impede the generation and the suppressive function of IL-10-producing Type 1 regulatory (Tr1) cells and CD25^+^Foxp3^+^ regulatory T (Treg) cells in the periphery (8–11). All these findings indicate that OX40L induced by TSLP serves as the functionally dominant molecule in initiating and amplifying the downstream immunological cascade, which consequently gives rise to dysregulated inflammatory Th2 responses. Investigation into the molecular mechanism revealed that TSLP induces sustained nuclear translocation of p50 and RelB, which form a transcriptionally active complex that binds to the κB-like sequences of *OX40L* promoter and induces OX40L expression by mDCs (12). The unique inflammatory Th2-polarizing property of surface OX40L expressed by TSLP-mDCs not only exemplifies the functional plasticity of DCs in inducing distinct T helper cell responses, but also underscores the critical role of DCs in the pathogenesis of allergic diseases (13–16).

C-type lectin receptors (CLRs) expressed by DCs can not only facilitate antigen uptake, but also determine and regulate downstream T cell responses (17–21). Dectin-1 is a unique CLR that upon ligation activates both canonical and non-canonical NF-κB pathways for cytokine expression, which induces Th1 and Th17 cell responses that are essential to host defense against fungi and mycobacteria (20, 22). In addition, Dectin-1 can crosstalk with other pattern recognition receptors (PRRs) including Toll-like receptor (TLR) and complement receptor, which not only quantitatively alters the magnitude of immune response, but also qualitatively modifies it (18, 22, 23). Therefore, we speculate that modulation of DC’s phenotype and function *via* Dectin-1 could regulate the undesired allergic Th2 cell responses driven by TSLP-NF-κB-OX40L axis.

In this study, we report that activation of human mDCs *via* Dectin-1 by agonistic ligand β-(1, 3)-glucan curdlan or anti-hDectin-1-Pam_3_CSK_4_ conjugate inhibited TSLP-induced OX40L expression. Along with the phenotypic change, Dectin-1 stimulation resulted in a distinct cytokine profile of TSLP-mDCs by inducing IL-10 production, which contributed to the downregulation of OX40L expression *via* STAT3 activation in an autocrine manner. In addition, activation of mDCs *via* Dectin-1 not only impaired the formation of p50-RelB complex but also suppressed the binding of p50 and RelB to *OX40L* promoter, which consequently inhibited TSLP-induced OX40L expression. Diminished OX40L expression by mDCs impaired generation of inflammatory Th2 cell response as well as maintenance of CRTH2^+^ memory CD4^+^ T cells. Dectin-1 stimulation also inhibited TSLP-induced STAT6 activation and production of Th2-attracting chemokine, CCL17, by mDCs. Such potent ability of Dectin-1 in suppressing Th2-type inflammatory responses was further demonstrated in allergy patients *ex vivo* and non-human primates (NHPs) *in vivo*.

## Materials and Methods

### Primary Human Cell Culture

Blood samples from healthy donors and allergy patients were acquired in accordance with the protocols approved by the Institutional Review Board (IRB). Human peripheral blood mononuclear cells (PBMCs) were isolated from buffy coat of healthy donors by density gradient centrifugation using Ficoll-Paque PLUS (GE Healthcare). Blood DCs were pre-enriched using EasySep Human Pan-DC Pre-Enrichment Kit (Stemcell Technologies) and myeloid DCs (mDCs, Lin1^-^HLA-DR^+^CD11c^+^CD123^-^) were then sorted by BD FACSAria (purity>99%). CD4^+^ T cells were enriched from PBMCs using EasySep Human CD4^+^ T Cell Enrichment Kit (Stemcell Technologies). Naïve CD4^+^ T cells (CD4^+^CD45RA^+^CD45RO^-^CCR7^+^) or CRTH2^+^ memory CD4^+^ T cells (CD4^+^CD45RA^-^CD45RO^+^ CRTH2^+^) were then sorted by BD FACSAria (purity >99%).

PBMCs from blood samples of allergy patients (n = 8) were isolated by density gradient centrifugation as mentioned above. All patients (**Table S1**) recruited in this study showed allergic response to ragweed pollen in skin prick test.

L cells and OX40L-transfected L cells (OX40L-L cells) (24) were maintained in cell culture medium supplemented with 600 ng/ml geneticin (Gibco). Complete RPMI (cRPMI) medium consisted of RPMI 1640 (Gibco) supplemented with 25 mM HEPES, 2 mM L-glutamine, 100 μM nonessential amino acids, 1 mM sodium pyruvate, 100 units/ml penicillin and 100 μg/ml streptomycin. Cell culture medium consisted of cRPMI and 10% heat-inactivated GemCell fetal bovine serum (Gemini Bio).

### DC Activation

FACS-sorted mDCs (2×10^5^/well) were incubated with 20 ng/ml TSLP (R&D Systems, 1398-TS-010/CF) (12), 100 μg/ml endotoxin-free curdlan (FUJIFILM Wako Pure Chemical Corporation) (24), 20 μg/ml (133 nM) anti-human Dectin-1 monoclonal antibody (anti-hDectin-1 mAb, clone 15E2) (18), 133 nM Pam_3_CSK_4_ (Invitrogen) or 133 nM anti-hDectin-1-Pam_3_CSK_4_ conjugate (described below) for indicated time. Cytokine concentration in the supernatants was measured by a bead-based multiplex immunoassay (Bio-Rad) according to manufacturer’s instruction. In cytokine blocking experiments, 10 μg/ml anti-IL-10 (BioLegend, 506813), anti-IL-10R (BioLegend, 308817), isotype control rat IgG2a (BioLegend, 400543), anti-TNFα (R&D Systems, MAB210-100) or isotype control mouse IgG1 (BioLegend, 400166) was added into culture 1h or 3h before stimulation. In some experiments, 1 μg/ml recombinant human TNFα (R&D Systems, 210-TA/CF) or IL-10 (PeproTech, 200-10) was added into culture. For inhibition assay, 10 μM STAT3 inhibitor Stattic (25) (Sigma-Aldrich, S7947-25MG), STA-21 (26) (Cayman Chemical, 14996) or Syk inhibitor R406 (24, 27) (Invivogen, inh-r406) was used to inhibit kinase activity as indicated. DMSO was used as vehicle control at 0.1%.

### T Cell Assay

After 24h incubation with TSLP, curdlan or anti-hDectin-1-Pam_3_CSK_4_ conjugate, mDCs (5×10^3^/well) were cocultured with FACS-sorted autologous CRTH2^+^ memory CD4^+^ T cells or allogeneic naive CD4^+^ T cells (1×10^5^/well). DCs were not washed before mixing with CD4^+^ T cells. In some experiments, anti-OX40L mAb (10 μg/ml, clone 19A3) (11) or isotype control mouse IgG2b (BioLegend, 400348) was added to mDCs 3h before coculture with allogeneic naive CD4^+^ T cells. On day 7 of coculture, CD4^+^ T cells were restimulated with PMA/ionomycin for 6h in the presence of GolgiPlug and GolgiStop (BD), followed by intracellular staining of cytokines using Fixation/Permeabilization Solution Kit (BD). In parallel experiments, after 5 days of coculture, CD4^+^ T cells were restimulated with immobilized anti-CD3/CD28 (Gibco, 11161D) for 48h, as per the manufacturer’s instructions. Cytokines in the culture supernatants were assessed by a bead-based multiplex immunoassay. In some experiments, γ-irradiated OX40L-L cells or L cells (1×10^5^/well) were seeded first, and then mDCs and allogeneic naive CD4^+^ T cells were cocultured for 7 days, followed by intracellular staining of cytokines. To assess allergen-specific CD4^+^ T cell responses, allergy patient PBMCs were incubated with TSLP and/or curdlan for 3h before being loaded with 2 μg/ml Amb a 1 protein (generated in-house by Dr. Robert Coffman) and cultured for 7 days. Cells were then restimulated with 2 μg/ml Amb a 1 protein and 2 μM Amb a 1-derived peptide (from Dr. Robert Coffman), culture supernatants were harvested 48h after Amb a 1 restimulation and cytokines were measured by a bead-based multiplex immunoassay (Bio-Rad).

### Flow Cytometry Analysis

Anti-Human Lineage Cocktail 1-FITC (Lin1, 340546), anti-HLA-DR-APC/Cy7 (Clone L243, 335796), anti-CD11c-V450 (Clone B-ly6, 560369), anti-CD123-PE/Cy5 (Clone 9F5, 551065), anti-CRTH2-V450 (Clone BM16, 561661), anti-CD45RO-PE/Cy5 (Clone UCHL1, 555494), anti-CD45RA-FITC (Clone HI100, 555488), anti-CCR7-PE (Clone 3D12, 552176), anti-CD3-PE-CF594 (Clone UCHT1, 562280), anti-CD4-APC/Cy7 (Clone RPA-T4, 557871), anti-OX40L-PE (Clone ik-1, 558164), Goat anti-mouse IgG-FITC (349031) from BD Biosciences, anti-HLA-DR-Alexa Fluor 700 (Clone L243, 307626), CD123-PerCP/Cy5.5 (Clone 6H6, 306016), anti-CD1c-APC/Cy7 (Clone L161, 331520), anti-CD141-Brilliant Violet 785 (Clone M80, 344116), anti-CLEC10A (Clone H037G3, 354706), anti-CD40L-PE (Clone 24-31, 310806), anti-CD86-FITC (Clone BU63, 374204), anti-TSLPR-APC (Clone 1B4, 322808) and anti-IL-7Rα-PE/Cy5 (Clone A019D5, 351324) from BioLegend, Live/Dead Aqua and CFSE from Invitrogen were used for cell surface staining. Anti-IL-4-BV605 (Clone MP4-25D2, 500828), anti-IL-5-APC (Clone TRFK5, 504306), anti-IL-10-PE (Clone JES3-9D7, 501404), anti-IL-13-PerCP/Cy5.5 (Clone JES10-5A2, 501911), anti-IL-17-BV711 (Clone BL168, 512328), anti-IFNγ-BV785 (Clone 4S.B3, 502542), anti-P-STAT3 (Tyr705)-Alexa Fluor 647 (Clone 13A3-1, 651007), mouse IgG1-Alexa Fluor 647 (Clone MOPC-21, 400155) from BioLegend were used for intracellular staining. Anti-CD3-V450 (Clone SP34-2, 560352), anti-CD11c-Alexa Fluor 647 (Clone 3.9, 565912), anti-CD14-FITC (Clone M5E2, 561712) from BD Biosciences were used to stain blood cells of rhesus macaque. Cell surface and intracellular flow cytometry staining was conducted according to manufacturer’s instruction. Flow cytometry data were acquired on BD LSRFortessa system. Live singlets were gated for analysis using FlowJo software and gates for expression of surface markers and intracellular cytokines were made based on isotype controls.

### ELISA for CCL17

The amount of chemokine CCL17 in mDC culture supernatants was measured using Human CCL17/TARC ELISA kit (R&D Systems, DY364) according to manufacturer’s instruction.

### Co-Immunoprecipitation

After 40h incubation with indicated stimuli, mDCs were washed with ice-cold PBS and lysed using NE-PER Nuclear and Cytoplasmic Extraction Reagents kit (Thermo Scientific, 78833). Halt Protease and Phosphatase Inhibitor Cocktail (Thermo Scientific, 78442) was used according to the manufacturer’s instructions. Protein concentrations of cytoplasmic and nuclear extracts were measured using Pierce BCA Protein Assay Kit (Thermo Scientific, 23227). Nuclear proteins of mDCs (25 μg/group) were pre-cleared by incubation with Protein A/G Magnetic Beads (Thermo Scientific, 88802) for 1h with continuous rotation at 4°C. After removal of beads, nuclear proteins were incubated with anti-p105/p50 (Cell Signaling Technology, 13586S) or anti-RelB (Abcam, ab33917) and Protein A/G Magnetic Beads overnight with continuous rotation at 4°C. Beads were then harvested, followed by extensive wash. Immunoprecipitants were eluted and denatured by heating with SDS-Sample Buffer (Boston BioProducts, BP-111R) at 90°C for 5 minutes. Proteins were then separated by electrophoresis in SDS/PAGE and transferred to PVDF membrane (Bio-Rad, 1620177) for immunoblotting.

### Western Blot

Western blot was performed as previously reported (27). The following antibodies were used for immunoblotting: OX40L (14991S), p105/p50 (13681S), p65 (6956S), RelB (10544S), c-Rel (67489S), HDAC1 (5356S), HSP90 (4877S), P-STAT3 (Tyr705, 9145S), STAT3 (12640S), P-STAT6 (Tyr641, 56554S), STAT6 (5397S), β-Actin-HRP (5125S), Goat anti-Rabbit IgG-HRP (7074S), Horse anti-Mouse IgG-HRP (7076S) from Cell Signaling Technology; p100/p52 (05–361) from Millipore; StarBright Blue 700 Goat anti-Mouse IgG (12004159), StarBright Blue 520 Goat anti-Rabbit IgG (12005870) from Bio-Rad; VeriBlot-HRP (ab131366) from Abcam. Images were acquired and analyzed using Bio-Rad ChemiDoc MP Imaging System.

### NF-κB Activation Assay

Nuclear extracts of mDCs were prepared after 24h stimulation with indicated reagents. NF-κB activity was determined by measuring DNA binding of NF-κB family members using TransAM NF-κB family kit (Active Motif, 43296) according to the manufacturer’s instructions.

### ChIP and qPCR assay

ChIP assay was performed as previously reported (27). Anti-p50/p105 (13586S), anti-p65/RelA (8242S) and anti-RelB (10544S) were used for immunoprecipitation. Primers for the detection of precipitated DNA by polymerase chain reaction (PCR) assay were designed from the *OX40L* surrounding region of κB-like sequences as follows: 5’-CCTGTTAGCCCAGAGGAAAA-3’ and 5’-CCAGGGCCAGAGATAAAAGG-3’ (12). In some experiments, the expression of *OX40L* in mDCs was measured by quantitative PCR assay. *OX40L* forward primer 5’-TCACCTACATCTGCCTGCACTT-3’, reverse primer 5’-GAAACCTTTCTCCTTCTTATATTCGGTA-3’ (28). *GAPDH* forward primer 5’-GTCTCCTCTGACTTCAACAGCG-3’, reverse primer 5’-ACCACCCTGTTGCTGTAGCCAA-3’.

### HEK-Blue TLR2 Reporter Assay

Human TLR2-expressing HEK 293 cells (Invivogen) were incubated with anti-hDectin-1 mAb, Pam_3_CSK_4_, or anti-hDectin-1-Pam_3_CSK_4_ conjugate at indicated concentrations for 16h. Response was detected by reading the optical density (OD) at 655 nm according to manufacturer’s instruction.

### Anti-hDectin-1-Pam_3_CSK_4_ Conjugate

The anti-hDectin-1-Pam_3_CSK_4_ conjugate was prepared by a Staudinger reaction between an azide-modified lipopeptide and an antibody modified with an ortho-phosphinoester (**Figure S1**) (29). First, the antibody was modified by reaction with 10 equivalents of an NHS-substituted Staudinger linker (10% DMSO in PBS, 4°C, 2 mg/ml protein concentration). Unreacted linker was quenched by addition of excess Tris and the modified protein purified by dialysis against PBS (10kDa MWCO dialysis cassette). Similarly, Pam_3_CSK_4_ was prepared for conjugation by reaction with an NHS-substituted PEG (3500) azide (1 eq in PBS, 0°C for 2h). Three equivalents of the resulting azide-modified Pam_3_CSK_4_ were then added to the Staudinger-labeled anti-hDectin-1 and the mixture allowed to react for 24h (0°C; PBS). The resulting anti-hDectin-1-Pam_3_CSK_4_ conjugate was then purified by extensive dialysis against PBS (10kDa MWCO).

### *In Vivo* Experiment on Non-human Primates

Two sets of *in vivo* experiments were performed using adult rhesus macaques (*Macaca mulatta*, body weight 9-12 kg and age 5-11 years of age). In the first experiment, as a pilot experiment, 5 male animals were sensitized by subcutaneous (SC) injection of 25 μg HDM (*Dermatophagoides farinae*) extract (Greer Labs) in aluminium hydroxide (alum) at two sites (12.5 μg HDM in 10 mg alum for each site), with DTaP injected intramuscularly (IM) as adjuvant (30). All animals received booster injections of 25 μg HDM in alum on weeks 3 and 5. As a control experiment, all five animals received SC injection of 25 μg HDM in PBS on weeks 6, 7, and 8. On weeks 10, 11, and 12, the same animals received SC injection of 25 μg HDM in PBS and intravenous (IV) injection of 1 mg anti-hDectin-1-Pam_3_CSK_4_ conjugate. Blood samples were collected at indicated time points and were used to measure Der p 1-specific serum IgE level by an ELISA assay (30).

In the second experiment, 7 female animals were sensitized by SC injection of 25 μg HDM extract in alum at two sites and IM injection of DTaP vaccine. All animals received booster injections of 25 μg HDM in alum on weeks 3, 5, 7, 9 and 11. For the control group, 4 animals then received SC injection of 25 μg HDM in PBS on weeks 12, 13, 14. For the treatment group, 3 animals received SC injection of both 25 μg HDM in PBS and 1 mg anti-hDectin-1-Pam_3_CSK_4_ conjugate and IV injection of 1 mg anti-hDectin-1-Pam_3_CSK_4_ conjugate. Blood samples were collected at indicated time points and Der p 1-specific serum IgE level was measured by ELISA. All aspects of animal work were performed in accordance with institutional guidelines for the California National Primate Research Center and approved by Primate Services Committee at the California Primate Research Center for animal handling, care and coordination, and veterinary care.

### Statistics

All figures with pooled data are represented as mean ± SD unless otherwise indicated. Statistical significance was determined using the analysis of variance (ANOVA) and paired *t* test with Prism 8 software (GraphPad Software) as indicated. Significance was set at *p* < 0.05. *, *p* < 0.05; **, *p* < 0.01; ***, *p* < 0.001; ****, *p* < 0.0001; ns, not significant.

## Results

### Dectin-1 Activation Inhibits TSLP-Induced OX40L Expression by mDCs

**Figure 1:**
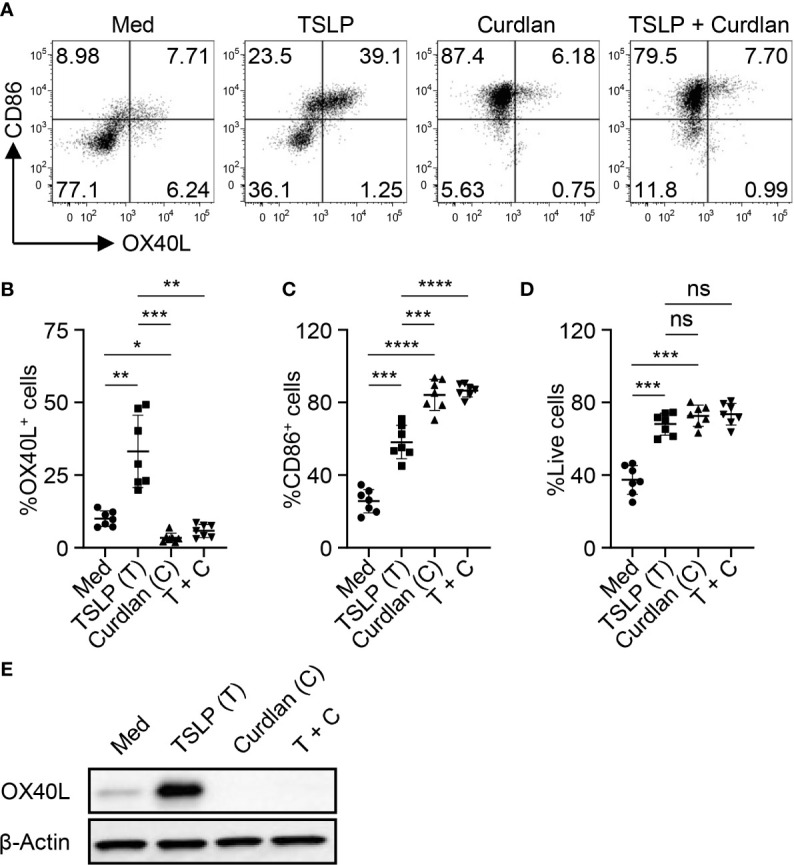
Dectin-1 Activation Suppresses TSLP-Induced OX40L Expression by mDCs and Th2 Cell Priming. **(A–C)** Sorted mDCs were incubated with medium (Med), TSLP and/or curdlan for 48h before surface staining of CD86 and OX40L. Live singlets were gated for analysis. Representative FACS data **(A)** and summarized data **(B, C)** of OX40L and CD86 expression from different donors (n = 7) are shown. **(D)** After 48h culture of sorted mDCs treated as in **(A)**, frequency of live cells in total cells was assessed by Live/Dead Aqua staining. Summarized data from different donors (n = 7) is shown. **(E)** Whole cell lysates of mDCs treated as in **(A)** were used for immunoblotting of OX40L. The data shown is representative of three independent experiments. Data in **(B, C)** are represented as mean ± SD. Significance was determined using one-way ANOVA with Tukey multiple comparisons test for **(B–D)**. *p < 0.05, **p < 0.01, ***p < 0.001, ****p < 0.0001 for the comparison between groups. ns, not significant.

We first tested whether Dectin-1 stimulation could regulate TSLP-induced OX40L expression by mDCs. Consistent with previous studies (5, 31), TSLP, when compared with medium alone, activated mDCs and potently induced surface OX40L expression, whereas Dectin-1 stimulation with curdlan further activated mDCs, yet suppressed TSLP-induced OX40L expression (**Figures 1A–C**). Treatment of mDCs with either curdlan or TSLP also enhanced their viability (**Figure 1D**). Immunoblotting of OX40L using whole cell lysates of mDCs (**Figure 1E**) confirmed our observation in **Figure 1A** and further suggested that Dectin-1-mediated suppression of surface OX40L expression is presumably by the reduction of total OX40L protein expression, but not *via* other mechanisms, including OX40L protein transportation to plasma membrane of mDCs. In addition, we also found that curdlan was capable of suppressing OX40L expression by TSLP-mDCs when mDCs encountered TSLP first, followed by curdlan in a sequential manner (**Figure S2**).

As previously reported (32), blood mDCs were further divided into 3 different subsets (**Figure S3**), CD1c^-^CD141^+^ mDCs (cDC1s), CD141^-^CD1c^+^CLECL10A^-^ mDCs (CLEC10A^-^ cDC2s) and CD141^-^CD1c^+^CLEC10A^+^ mDCs (CLEC10A^+^ cDC2s). Approximately 10% of blood mDCs are CD141^+^CD1c^-^ cDC1, the rest of them are CD141^-^CD1c^+^ cDC2. Within CD141^-^CD1c^+^ cDC2 population, about 10% to 20% are CD1c^hi^CLEC10A^+^, which have been reported to be pro-inflammatory cDC2s (32). As shown in **Figure S3B**, TSLP enhanced OX40L expression on both cDC1s and CLEC10A^-^ cDC2s, but not CLEC10A^+^ cDC2s. Curdlan treatment resulted in the suppression of TSLP-induced OX40L on both cDC1s and CLEC10A^-^ cDC2s. Although TSLP did not increase OX40L expression on CLEC10A^+^ cDC2s, curdlan could still downregulate OX40L expression.

Along with diminished OX40L expression, mDCs treated with TSLP and curdlan impaired TSLP-mDC-mediated Th2-type CD4^+^ T cell priming (**Figure S4**), and more importantly, curdlan-mediated suppression of Th2 response was dependent on OX40L (**Figure S5**). Taken together, activation of mDCs *via* Dectin-1 suppressed TSLP-induced OX40L expression followed by decreased Th2-type CD4^+^ T cell priming.

### Activation of mDCs *via* Dectin-1 Controls CRTH2^+^ Memory CD4^+^ T Cell Response

In addition to Th2 cell priming, TSLP-mDCs preferentially induce robust expansion of CRTH2^+^CD4^+^ T cells while maintain their central memory phenotype and Th2 commitment for rapid relapse of acute allergic inflammation upon re-exposure to allergen (7). We therefore investigated whether activation of TSLP-mDCs *via* Dectin-1 could also regulate CRTH2^+^ memory CD4^+^ T cell response.

**Figure 2:**
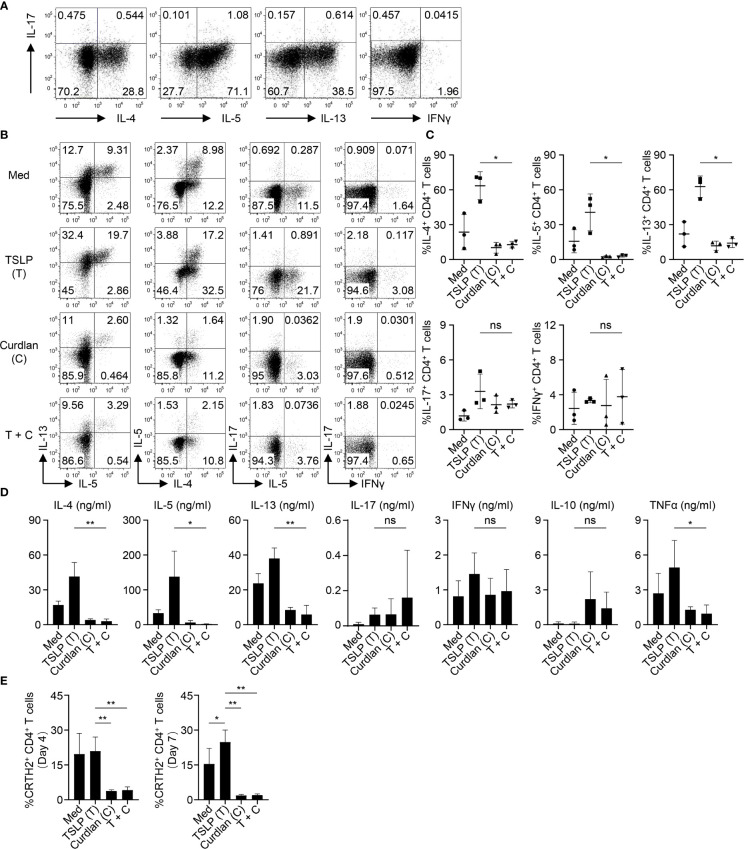
Activation of mDCs *via* Dectin-1 Inhibits TSLP-Induced Inflammatory Th2 Response of CRTH2^+^ Memory CD4^+^ T Cells. **(A)** Intracellular staining of cytokines expressed by sorted CRTH2^+^ memory CD4^+^ T cells upon *ex vivo* stimulation with PMA/ionomycin. The FACS data shown is representative of three independent experiments. **(B, C)** Sorted mDCs were treated with indicated reagents for 24h before coculture with autologous CRTH2^+^ memory CD4^+^ T cells for 7d followed by intracellular staining of cytokines expressed by T cells. Live singlets were gated for analysis. Representative FACS data **(B)** and summarized data **(C)** from different donors (n = 3) are shown. **(D)** After 5d coculture of mDCs and autologous CRTH2^+^ memory CD4^+^ T cells as in **(B)**, T cells were stimulated by anti-CD3/28 beads for 48h before measuring cytokine production in culture supernatants by multiplex assay. Summarized data from different donors (n = 3) are shown. **(E)** After 4d or 7d of coculture of mDCs and autologous CRTH2^+^ memory CD4^+^ T cells as in **(B)**, surface CRTH2 expression on CD4^+^ T cells was measured by FACS staining. Summarized data from different donors (n = 3) are shown. Data in **(C–E)** are represented as mean ± SD. Significance was determined using one-way ANOVA with Tukey multiple comparisons test for **(C–E)**. *p < 0.05, **p < 0.01 for the comparison between groups. ns, not significant.

We first confirmed the nature of CRTH2^+^ memory CD4^+^ T cells purified from peripheral blood of healthy donors by assessing their ability to express Th2 cytokines (IL-4, IL-5 and IL-13) upon T cell activation *ex vivo* (**Figure 2A**). Almost no or low levels of Th1 cytokine (IFNγ) or Th17 cytokine (IL-17A) expression was observed. This is consistent with previous studies that CRTH2^+^CD4^+^ T cells represent the committed Th2 cells *in vivo* (33, 34). CRTH2^+^ memory CD4^+^ T cells were cultured with autologous mDCs activated by TSLP and/or curdlan. When compared with mDCs cultured in medium alone, TSLP-mDCs promoted Th2 response as evidenced by increased frequencies of IL-4-, IL-5- and IL-13-expressing T cells (**Figures 2B, C**). Treatment with curdlan, even in the presence of TSLP, instructed mDCs to preferentially suppress type 2 cytokine expression by T cells (**Figures 2B, C**). This was further confirmed by measuring cytokines secreted in the culture supernatants in parallel experiments (**Figure 2D**). More importantly, curdlan overturned TSLP-mDC-induced inflammatory property of CRTH2^+^ memory CD4^+^ T cells, as evidenced by the decreased expression of TNFα, and a trend of enhanced IL-10 production, which varied among different donors (**Figure 2D**).

CRTH2 serves as the receptor for pro-inflammatory factor prostaglandin D2 (PGD2), which is mainly produced by mast cells at the site of allergic inflammation (35). CRTH2 therefore selectively mediates chemotactic migration of blood CRTH2^+^ Th2 cells towards PGD2 (35). In addition, interaction between PGD2 and CRTH2 preferentially induces type 2 cytokine production and prevents the apoptosis of Th2 cells (36, 37). TSLP-mDCs have been reported to maintain CRTH2 expression by Th2 cells, which is known to decrease following T cell activation (7). We found that activation of mDCs *via* Dectin-1 impaired TSLP-mediated maintenance of CRTH2 expression by T cells on both day 4 and day 7 of mDC-T cell coculture (**Figure 2E**), suggesting that Dectin-1 stimulation decreased the capacity of TSLP-mDCs in maintaining the functional phenotype and Th2 commitment of CRTH2^+^ memory CD4^+^ T cells.

### Dectin-1-Induced IL-10 Expression and STAT3 Activation Result in Suppression of OX40L Expression on TSLP-mDCs

**Figure 3:**
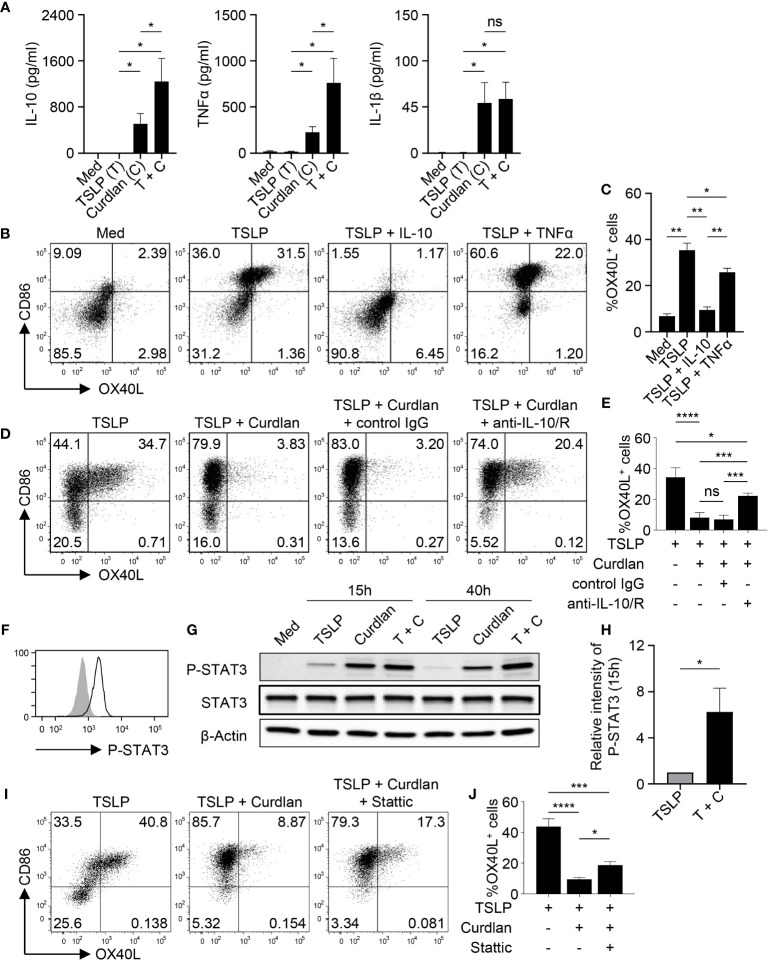
IL-10 Downregulates TSLP-Induced OX40L in an Autocrine Manner. **(A)** Cytokine expression by mDCs (n = 4) upon indicated stimulation was analyzed by multiplex assay. **(B, C)** Sorted mDCs were treated indicated reagents for 48h before surface staining of CD86 and OX40L. Representative FACS data **(B)** and summarized data **(C)** from different donors (n = 4) are shown. **(D, E)** Sorted mDCs were pre-incubated with anti-IL-10 and anti-IL-10R, or isotype control before stimulation with TSLP and/or curdlan for 48h followed by surface staining of CD86 and OX40L. Representative FACS data **(D)** and summarized data **(E)** from different donors (n = 4) are shown. **(F)** STAT3 phosphorylation (open histogram) was detected in mDCs treated with TSLP and curdlan for 48h. Tinted histogram denotes isotype control. Representative FACS data of three independent experiments is shown. **(G, H)** Treatment with TSLP plus curdlan, when compared with TSLP alone, enhanced STAT3 phosphorylation in mDCs. Representative immunoblot **(G)** and summarized data **(H)** of different donors (n = 3) upon treatment for indicated time are shown. The intensity of P-STAT3 was first normalized to that of β-Actin in the same group, and then relative intensity was obtained by comparison with Med group. **(I, J)** Sorted mDCs were pre-incubated with STAT3 inhibitor Stattic for 3h before stimulation with TSLP and/or curdlan for 48h followed by surface staining of CD86 and OX40L. Representative FACS data **(I)** and summarized data **(H)** from different donors (n = 3) are shown. Data in **(A, C, E, H, J)** are represented as mean ± SD. Significance was determined using one-way ANOVA with Tukey multiple comparisons test for **(A, C, E, J)** and paired t test (two-tailed) for **(H)**. *p < 0.05, **p < 0.01, ***p < 0.001, ****p < 0.0001 for the comparison between groups. ns, not significant.

Given the critical role of OX40L in promoting Th2 responses, we then investigated the mechanism underlying the diminished OX40L expression by TSLP-mDCs upon Dectin-1 stimulation. As shown in **Figure 3A**, curdlan induced IL-10, TNFα and IL-1β expression while TSLP only induced these cytokines at background level. However, combined treatment with TSLP and curdlan induced even higher levels of IL-10 and TNFα than single stimulation (**Figure 3A**), suggesting that the intrinsic signaling cascades initiated by Dectin-1 and TSLP receptor could possibly crosstalk with each other for altered cytokine expression.

Given the substantial amount of IL-10 expression by mDCs upon TSLP and curdlan stimulation, we investigated its possible involvement in regulating OX40L expression. Indeed, exogenous recombinant IL-10 and TNFα differentially suppressed TSLP-induced OX40L expression (**Figures 3B, C**), and IL-10, when used at the same concentration, is much more potent than TNFα in suppressing TSLP-induced OX40L expression (**Figure 3C**), suggesting that IL-10 could be the major effector cytokine repressing OX40L expression in an autocrine way. Reversely, blocking IL-10 significantly restored OX40L expression by mDCs treated with TSLP plus curdlan (**Figures 3D, E**), which confirmed that IL-10 produced by mDCs upon Dectin-1 stimulation contributed to downregulating TSLP-induced OX40L expression in an autocrine way. Activation of STAT3 was observed in mDCs upon TSLP plus curdlan treatment (**Figure 3F**). Of note, when compared with TSLP alone, a combination of TSLP and curdlan resulted in much stronger and long-lasting (up to 40h) STAT3 phosphorylation (**Figures 3G, H**). To further investigate the role of STAT3 in regulating OX40L expression, we performed inhibition assay and found that inhibition of STAT3 activity partially restored OX40L expression (**Figures 3I, J** and **S6**), suggesting that STAT3 activation induced by Dectin-1 stimulation, in line with autocrine IL-10, contributed to the downregulation of OX40L expression.

### Dectin-1 Suppresses TSLP-Induced OX40L Expression by Inhibiting p50 and RelB Activity

Blocking IL-10 signaling substantially but not fully restored OX40L expression by mDCs treated with TSLP plus curdlan (**Figures 3D, E**), suggesting that Dectin-1 could repress TSLP-induced OX40L expression *via* mechanism other than autocrine IL-10. When Syk activity was inhibited, OX40L expression by mDCs treated with TSLP plus curdlan was almost fully restored (**Figure S6**), suggesting that Dectin-1/Syk signaling cascade is indispensable for inhibition of TSLP-induced OX40L. TSLP receptor complex is a heterodimer that consists of a common γ-like subunit called TSLP receptor (TSLPR) and an IL-7 receptor α chain (IL-7Rα) (38, 39). We measured the expression of both subunits on mDCs upon different treatments (**Figure S7**) and concluded that decreased OX40L expression upon Dectin-1 stimulation is unlikely due to reduced expression of TSLP receptor complex for signaling transduction. We also observed that Dectin-1 stimulation did not alter IL-10Rα expression on TSLP-mDCs (data not shown).

**Figure 4:**
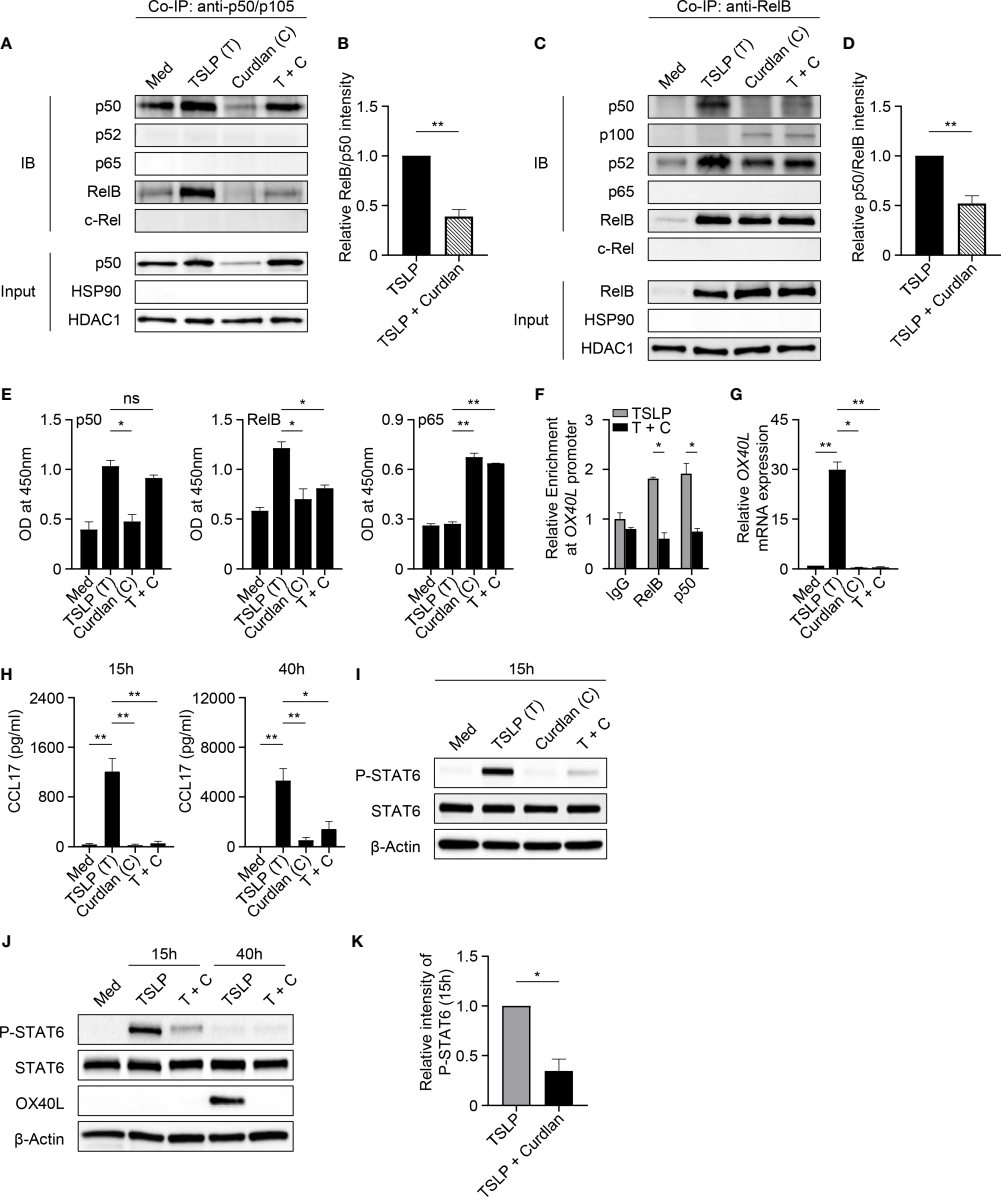
Dectin-1 Stimulation Inhibits TSLP-Induced OX40L and CLL17 Expression. **(A–D)** Nuclear proteins from mDCs treated with Med, TSLP and/or curdlan for 40h were used for Co-IP assay. Anti-p50/p105 **(A, B)** or anti-RelB **(C, D)** was used as pull-down antibody. Proteins in the immunoprecipitants were analyzed by immunoblotting (IB). In input controls of nuclear proteins, HDAC1 was probed as loading control and HSP90 was probed to investigate the possible cytoplasmic contamination. Representative Western blot data **(A, C)** and summarized data **(B, D)** from three independent experiments are shown. The intensity of co-immunoprecipitated protein RelB **(A, B)** or p50 **(C, D)** was first normalized to that of IP protein p50 **(A, B)** or RelB **(C, D)** in immunoprecipitants, and then relative intensity in TSLP + Curdlan (T + C) group was obtained by comparison with TSLP group. **(E)** DNA binding of NF-κB subunits in nuclear extracts of mDCs from different donors (n = 3) treated with indicated reagents was measured by ELISA. **(F)** ChIP assay demonstrates the relative enrichment of RelB and p50 at the region around the κB-like sequences of OX40L promoter in response to TSLP or TSLP and curdlan (T + C). Summarized data from three independent experiments are shown. **(G)** Quantitative PCR for OX40L mRNA in mDCs from different donors (n = 3) after incubation with indicated reagents for 40h. OX40L expression is normalized to GAPDH and set at 1 in mDCs cultured in Med. **(H)** CCL17 in culture supernatants of mDCs (n = 4) after incubation with indicated reagents for 15h or 40h was measured by ELISA. **(I–K)** Whole cell lysates of mDCs incubated with indicated reagents for 15h or 40h were used for immunoblotting. Representative Western blot data **(I, J)** and summarized data **(K)** from different donors (n = 3) after 15h stimulation are shown. The intensity of P-STAT6 was first normalized to that of STAT6 in the same group, and then relative intensity was obtained by comparison with Med group. Data in **(B, D–H, K)** are represented as mean ± SD. Significance was determined using paired t test (two-tailed) for **(B, D, K)** and one-way ANOVA with Tukey multiple comparisons test for **(E, G, H)**, and two-way ANOVA with Tukey multiple comparisons test for **(F)**. *p < 0.05, **p < 0.01 for the comparison between groups. ns, not significant.

We next investigated whether Dectin-1 stimulation could affect the activities of p50 and RelB, which are required for *OX40L* transcription in TSLP-mDCs (12). In Co-IP experiments using mDC nuclear proteins, when p50 was pulled down, lower level of co-immunoprecipitated RelB was detected in TSLP plus curdlan group in comparison to TSLP group (**Figures 4A, B**). Consistently, in the reverse Co-IP in which RelB was pulled down, lower level of co-immunoprecipitated p50 was probed in TSLP plus curdlan group when compared to TSLP group (**Figures 4C, D**), which confirmed that p50-RelB complex induced by TSLP was decreased upon Dectin-1 stimulation. We also found that RelB selectively formed complex with p100 in the nucleus of mDCs treated with curdlan alone, or TSLP plus curdlan (**Figure 4C**). Consistent with restored OX40L expression by mDCs (**Figure S6**), inhibition of Syk activity abrogated nuclear p100 accumulation in mDCs upon treatment with TSLP plus curdlan (**Figure S8**), while blocking IL-10 or TNFα partially reduced it, suggesting that Dectin-1/Syk signaling cascade and autocrine cytokines are involved in nuclear p100 accumulation. Previous studies reported that activation of NF-κB pathway by specific receptors including CD27, TNF receptor and lymphotoxin-β receptor results in p100 accumulation in nucleus (40, 41), and nuclear p100 can complex with RelB, which inhibits the transcription of NF-κB target genes (41). We therefore further investigated the transcriptional activity of RelB and found that curdlan suppressed DNA binding of RelB and enhanced that of p65 in TSLP-mDCs (**Figure 4E**). More importantly, in ChIP assay, we found that TSLP-induced binding of p50 and RelB to *OX40L* promoter was impaired by curdlan (**Figure 4F**), which is consistent with reduced *OX40L* expression at mRNA level (**Figure 4G**). Based on these observations, we concluded that Dectin-1 stimulation suppressed TSLP-induced OX40L expression by sequestering RelB in p100-RelB complex, therefore inhibiting the formation and transcriptional activity of p50-RelB complex.

### Dectin-1 Inhibits TSLP-Induced STAT6 Activation and CCL17 Production

TSLP activates mDCs to produce chemokine CCL17, which recruits CCR4-expressing Th2 cells into the originally inflamed tissue and further enhances Th2-mediated inflammation (1, 42). Indeed, we detected high amounts of CCL17 in the supernatant of TSLP-mDCs after 15h and 40h incubation (**Figure 4H**). However, curdlan, even in the presence of TSLP, induced a much lower production of CCL17 at both time points, suggesting that Dectin-1 stimulation suppressed TSLP-induced CCL17 production by mDCs. TSLP induces robust activation of JAK-STAT signaling pathway in mDCs (12). Specifically, STAT6 activated by TSLP binds to *CCL17* promoter for chemokine expression (12). Consistent with decreased CCL17 expression, Dectin-1 stimulation suppressed TSLP-induced STAT6 activation in mDCs as evidenced by decreased phosphorylation of STAT6 upon curdlan treatment (**Figures 4I–K** and **Figures S9A, B**) In addition, inhibition of Syk activity or blocking IL-10, but not TNFα, restored TSLP-induced P-STAT6 (**Figures S9C, D**), suggesting that Dectin-1 inhibits TSLP-induced phosphorylation of STAT6 *via* an Syk and IL-10-dependent manner.

### Dectin-1 Stimulation Inhibits Allergen-Specific Th2-Type T Cell Response in Allergy Patients *Ex Vivo*

**Figure 5:**
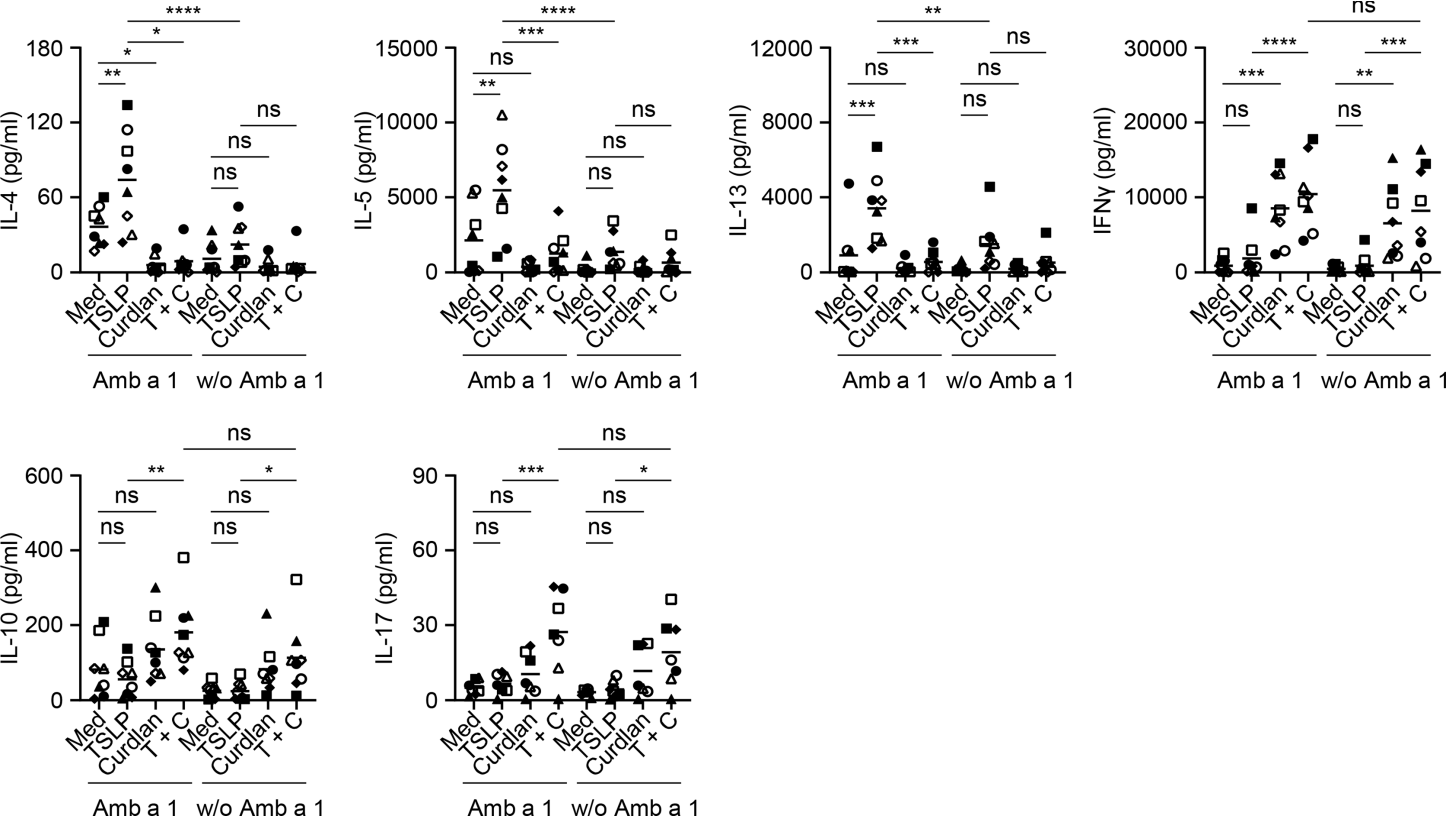
Dectin-1 Activation Inhibits TSLP-Induced Allergen-Specific Th2-Type T Cell Response. Cytokine production in culture supernatants with or without Amb a 1 restimulation was measured by multiplex assay. Summarized data from eight patients are shown. Data are represented as grand mean. Significance was determined using two-way ANOVA with Tukey multiple comparisons test. *p < 0.05, **p < 0.01, ***p < 0.001, ****p < 0.0001 for the comparison between groups. ns, not significant.

Common ragweed (*Ambrosia artemisiifolia*) represents one of the most prominent seasonal allergen sources in North America and parts of Europe and Asia (43, 44). Among several allergens described in ragweed pollen, Amb a 1 has been identified as the major allergen that is recognized by more than 90% of ragweed-sensitized individuals and accounts for more than 90% of the allergenic activity in ragweed pollen (45–47). We therefore tested the effectiveness of curdlan in suppressing allergen-specific Th2-type T cell response using Amb a 1 as model allergen. PBMCs isolated from allergy patients (**Table S1**), who showed allergic response to ragweed in the skin prick test, were treated with TSLP and/or curdlan in the presence of Amb a 1. Allergen-specific T cell responses were assessed by measuring cytokine production in the supernatants upon restimulation with Amb a 1 (**Figure 5**). In line with the data in **Figure 2**, TSLP enhanced Amb a 1-specific IL-4-, IL-5-, and IL-13-producing T cell responses. TSLP-induced Th2-type responses are mainly dependent on TSLP-induced OX40L expression on mDCs (5, 7). We further found that curdlan suppressed TSLP-induced allergen-specific Th2 cell response as evidenced by decreased Th2 cytokines including IL-4, IL-5 and IL-13. In addition, curdlan promoted the expression of IFNγ, IL-17 and IL-10 in the supernatant regardless of allergen restimulation, suggesting that curdlan suppressed allergen-specific Th2 response and promoted Th1 and Th17 response in a non-specific way.

### Anti-hDectin-1-Pam_3_CSK_4_ Conjugate Suppresses HDM-Specific Serum IgE Level in NHPs *In Vivo*

**Figure 6:**
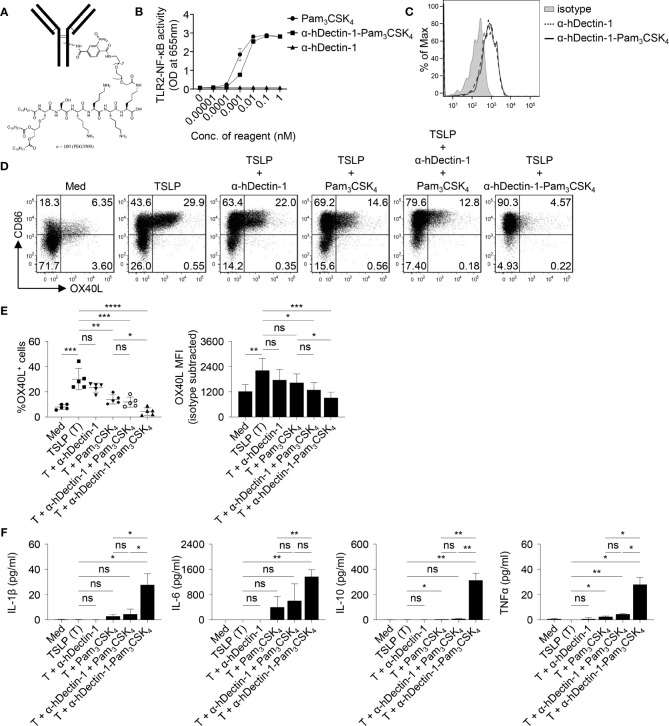
Anti-hDectin-1-Pam_3_CSK_4_ Conjugate Suppresses TSLP-Induced OX40L Expression by mDCs. **(A)** Diagram of anti-hDectin-1-Pam_3_CSK_4_ conjugate structure. **(B)** Anti-hDectin-1-Pam_3_CSK_4_ conjugate preserved TLR2 agonistic property in HEK-Blue TLR2 reporter assay. Summarized data of test with triplicate samples are shown. **(C)** Anti-hDectin-1-Pam_3_CSK_4_ conjugate bound to human mDCs similar to its mAb precursor. Representative FACS data of three experiments is shown. **(D, E)** Anti-hDectin-1-Pam_3_CSK_4_ conjugate suppressed TSLP-induced OX40L expression by human mDCs. Representative FACS data **(D)**, summarized data of % of OX40L^+^ mDCs (**E**, left panel) and mean fluorescence intensity (MFI) of OX40L expression (**E**, right panel) from different donors (n = 5) are shown. **(F)** Cytokine concentration in culture supernatants of mDCs (n = 4) after stimulation with indicated reagents was analyzed by multiplex assay. Data in **(B, E, F)** are represented as mean ± SD. Significance was determined using one-way ANOVA with Tukey multiple comparisons test for **(E, F)**. *p < 0.05, **p < 0.01, ***p < 0.001, ****p < 0.0001 for the comparison between groups. ns, not significant.

The effectiveness of Dectin-1 activation in controlling allergen-specific Th2 response was further tested in rhesus macaques *in vivo*. Given the heterogeneity and water insolubility of curdlan, we generated conjugates of anti-human Dectin-1 antibody (clone 15E2) and Pam_3_CSK_4_, a TLR2 ligand (**Figures 6A** and **S1**). We previously reported that anti-hDectin-1 antibody can synergize with Pam_3_CSK_4_ to activate DCs, resulting in enhanced IL-10 expression by DCs (18). The resulting anti-hDectin-1-Pam_3_CSK_4_ conjugate preserved TLR2 agonistic property (**Figure 6B**) and bound to human mDCs similarly to the precursor anti-hDectin-1 mAb (**Figure 6C**). Anti-hDectin-1-Pam_3_CSK_4_ conjugate was also capable of suppressing TSLP-induced OX40L expression by mDCs (**Figures 6D, E**). Indeed, anti-hDectin-1-Pam_3_CSK_4_ conjugate was more potent than anti-hDectin-1 mAb, Pam_3_CSK_4_, or a mixture of them at the same molar concentration. In addition, anti-hDectin-1-Pam_3_CSK_4_ conjugate activated mDCs to express cytokines including IL-10 (**Figure 6F**). These observations suggest that similar to curdlan (**Figure 1D**), anti-hDectin-1-Pam_3_CSK_4_ conjugate activated mDCs meanwhile suppressed TSLP-induced OX40L expression. Consistent with reduced OX40L expression, TSLP-mDCs treated with anti-hDectin-1-Pam_3_CSK_4_ conjugate resulted in decreased inflammatory Th2 response in naïve CD4^+^ T cell priming (**Figures S10** and **S11**).

**Figure 7:**
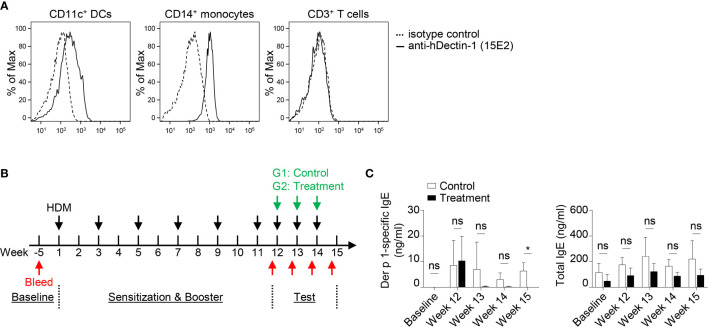
Anti-hDectin-1-Pam_3_CSK_4_ Conjugate Can Suppress Allergen-Specific IgE Response in Macaques *In Vivo.* **(A)** Anti-hDectin-1 mAb (clone 15E2) bound to CD11c^+^ DCs and CD14^+^ monocytes in macaque PBMCs. Representative FACS data of five animals is shown. **(B)** Scheme of the second in *vivo* test in NHP. Injection of HDM extract, bleeding, and treatment with anti-hDectin-1-Pam_3_CSK_4_ conjugate was performed as indicated. **(C)** Der p 1-specific IgE and total IgE in the serum of animals at indicated time points were measured by ELISA. Data in **(C)** are represented as mean ± SD. Significance was determined using two-way ANOVA with Tukey multiple comparisons test for **(C)**. *p < 0.05 for the comparison between groups. ns, not significant.

A previous study demonstrated that macaques sensitized with house dust mite (HDM, *Dermatophagoides farinae*) extract developed TSLP-mediated Th2 responses *in vivo* (48). OX40L-blocking antibody also inhibited HDM-specific IgE response (48). Anti-hDectin-1 antibody (Clone 15E2) generated in mice does not bind to murine Dectin-1 (data not shown). We thus tested the effectiveness of anti-hDectin-1-Pam_3_CSK_4_ in controlling HDM-specific IgE response in macaques. Before testing anti-hDectin-1-Pam_3_CSK_4_ conjugate in macaques, we confirmed that anti-hDectin-1 antibody (clone 15E2) bound to CD11c^+^ DCs and CD14^+^ monocytes, but not T cells, in the blood of macaques (**Figure 7A**). In the pilot experiment (depicted in **Figure S12A**), five animals were first sensitized and boosted with HDM extract, followed by a control stage in which all the animals were challenged with three doses of HDM. The same animals were then treated with three doses of anti-hDectin-1-Pam_3_CSK_4_ along with HDM challenge in a test stage. The HDM major allergen (49) Der p 1-specific IgE level in the serum was monitored at multiple time points as indicated by the experiment scheme (**Figure S12A**). We found that total serum IgE remained unchanged through the experiment (**Figure S12B**), however, Der p 1-specific IgE was found substantially, but not significantly, increased after HDM booster, which was then decreased to baseline level after anti-hDectin-1-Pam_3_CSK_4_ treatment (**Figure S12B**).

Given the variation in Der p 1-specific IgE level observed among different animals after HDM booster injection in the pilot experiment (**Figure S12B**), we therefore, in the second experiment (n = 7, **Figure 7B**), enhanced the booster procedure in addition to separating animals into two groups. All seven animals were sensitized and boosted five times with HDM. Animals in the control group (n = 4) were then challenged with HDM, while the test group (n = 3) received anti-hDectin-1-Pam_3_CSK_4_ treatment in addition to HDM challenge (**Figure 7B**). The total serum IgE remained similar between two groups (**Figure 7C**). In contrast, Der p 1-specific serum IgE was significantly lower in animals received anti-hDectin-1-Pam_3_CSK_4_ treatment when compared to controls at week 15, suggesting that anti-hDectin-1-Pam_3_CSK_4_ decreased allergen-specific type-2 immune response *in vivo*.

## Discussion

In this study, we presented both cellular and molecular mechanisms of Dectin-1 in controlling TSLP-induced inflammatory Th2 response associated with allergic disorders. TSLP plays an important role in the pathogenesis of allergic inflammation at the interface between epithelial cells and DCs (50, 51). Allergens and viruses can trigger epithelial cells to express TSLP. Indeed, TSLP is highly expressed by keratinocytes in skin lesions of patients with atopic dermatitis (1) and by airway epithelial cells of individuals with allergic asthma (3). Humanized mAb against TSLP has been reported to lower the rates of asthma exacerbations in clinical trials when compared with placebo (52, 53). OX40L expressed on TSLP-mDCs plays an essential role in the TSLP-induced inflammatory Th2 responses (5, 7). In addition to the induction of inflammatory Th2-type T cell differentiation, TSLP-mDCs elicit a robust expansion of memory Th2-type T cells, while maintaining their central memory phenotype and Th2 commitment (7). The interaction between OX40L and OX40 controls the fate of CD4^+^ T cells during allergic inflammation (54). Blockade of OX40L could thus result in the suppression of TSLP-driven atopic inflammation (48). Our findings in this study revealed that targeting Dectin-1 with agonists changed the phenotype of TSLP-mDCs by diminishing OX40L expression and simultaneously introducing IL-10 production, which has been reported to prevent airway hypersensitivity upon allergen exposure (55). More importantly, these changes on mDCs consequently impaired Th2 cell priming from naïve CD4^+^ T cells as well as Th2 commitment of CRTH2^+^ memory CD4^+^ T cells.

In patients with allergic diseases, Th2 cytokines play fundamental roles in the disease pathogenesis by inducing antibody isotype switching, activation of granulocytes, including eosinophilia, airway hyperactivity and mucus production (56). Although Th2-type CD4^+^ T cells are not the sole source of these cytokines, numerous studies have established their requirement for the hallmark features of allergic diseases, including allergic asthma (57). In addition, Th2 cytokines also act on epithelial cells to enhance their production of innate cytokines and augment the secretion of proinflammatory mediators by mast cells and basophils (57). Using ragweed pollen Amb a 1 and HDM extract as model allergens, we further provided evidence that Dectin-1 stimulation resulted in decreased type-2 immune response both *in vitro* and *in vivo* in an allergen-specific manner. In terms of HDM-mediated allergic responses, previous study by Chu et al. (58) reported that disrupting IL-33, but not TSLP or IL-25 signaling, reduced allergic responses in mice with short-term (10 days) sensitization to HDM. On the other hand, Chen et al. (59) reported anti-TSLP mAb inhibited Th2 inflammation in a mouse model with longer HDM exposure (up to 5 weeks). Though both studies used HDM as model allergen, difference in length/duration of allergen exposure is likely to explain the discrepancy in the conclusion: IL-33 is preferentially induced during the early stage of HDM exposure (58), thus plays a critical role in the HDM sensitization and initiation of allergy, while TSLP plays a non-redundant role in HDM-mediated allergic pathogenesis. A recent study reported that TSLP-activated CD11c^+^ DCs induce human T follicular helper (Tfh) cell differentiation *via* an OX40L-dependent way (31). Therefore, it will be valuable to investigate the effects of Dectin-1 targeting in regulating Tfh polarization in allergic and inflammatory disorders in future study.

At the molecular level, PU.1 and p50-RelB complex have been reported to mediate *OX40L* transcription in human DCs under homeostatic condition and upon activation, respectively (12, 60). We chose to investigate the formation and activity of nuclear p50-RelB complex in mDCs given that either curdlan or TSLP triggered NF-κB pathway and curdlan can further activate TSLP-mDCs, as evidenced by enhanced CD86 expression. Based on our Co-IP and ChIP results, we conclude that Dectin-1 stimulation induced p100-RelB complex in the nucleus, which sequestered RelB for p50-RelB formation and therefore inhibited *OX40L* transcription. We chose to use mDCs (0.1% to 0.5% in PBMCs) sorted from peripheral blood for these assays to avoid any misinterpretation derived from the use of monocyte-derived DCs generated *in vitro*. Our study therefore shows the feasibility of conducting comprehensive cell signaling studies using low-yield nuclear protein (~3 μg per 10^6^ cells) isolated from rare primary human DC population. A previous study performed with mouse bone marrow-derived DCs (BMDCs) showed that curdlan induced OX40L expression (61), which is inconsistent with our data in this study. It is important to note that they used mouse BMDCs generated with GM-CSF and IL-4 (61), whereas blood circulating human mDCs were used in our study. In addition to potential intrinsic differences between mouse and human DCs, we have also known that *in vitro* generated human monocyte-derived DCs (mo-DCs) do not express OX40L in response to TSLP (data not shown). Furthermore, curdlan suppressed OX40L expression by mDCs, but this was not the case for plasmacytoid DCs as we previously reported (24). It is also known that mouse BMDC cultures comprise a heterogenous population of cells (62) that may not respond to curdlan equally. Therefore, it is difficult to directly compare data generated with TSLP-treated human primary mDCs with those generated with mouse BMDCs without extra TSLP treatment. Another study also reported that soluble egg antigen (SEA) from *Schistosoma mansoni* that can bind to Dectin-1, induced OX40L expression on human mo-DCs (63). However, SEA can also bind to other receptors including DC-SIGN, mannose receptor, and macrophage galactose type-lectin (MGL), as described in the same report (63).

As an effort to translate our *in vitro* observations *in vivo*, we tested anti-hDectin-1-Pam_3_CSK_4_ conjugate in rhesus macaques. Curdlan has been widely used as a Dectin-1 agonist in this and other studies (22, 64). It has also been known that curdlan-induced DC activation is mainly *via* its biding to Dectin-1 (22, 24, 64). However, it might not be suitable to be used in clinics, partly due to its insolubility and potential quality control issues. We have tested phospho-curdlan, a soluble form, but it did not effectively suppress TSLP-induced OX40L expression by mDCs (data not shown). Anti-hDectin-1 (clone 15E2) is an agonistic antibody (18), but this antibody alone, as shown in this study, was not enough to suppress TSLP-induced OX40L expression. As we previously reported, however, anti-hDectin-1 can synergize with Pam_3_CSK_4_ to activate DCs to induce IL-10 and other cytokine expression (18). Therefore, we generated anti-hDectin-1-Pam_3_CSK_4_ conjugate as surrogate agonist. Anti-hDectin-1-Pam_3_CSK_4_ conjugate was further validated *in vitro* for its potential to suppress TSLP-induced OX40L expression by mDCs as well as subsequent inflammatory Th2 response. There are several limitations in our *in vivo* experiments using macaques, including a small number of animals and limited *in vivo* data that will need to be further validated in future. However, decrease of Der p 1-specific serum IgE upon anti-hDectin-1-Pam_3_CSK_4_ conjugate treatment supported our human *in vitro* data. This is also in line with previously published data showing that β-glucan receptor Dectin-1 was required for lung defense during acute, invasive *Aspergillus fumigatus* infection (65–67). Interestingly, it is also important to note that blocking Dectin-2, another lectin receptor, resulted in the reduction of cysteinyl leukotriene expression in response to *Aspergillus fumigatus* and HDM (68, 69) and could thus ameliorate allergic lung pathogenesis in mice.

Collectively, our study provided the mechanism of Dectin-1 in regulating Th2 inflammation in allergic disorders driven by TSLP-OX40L-DC axis. This study also provides us with a novel strategy to control Th2-type inflammatory response by manipulating functional plasticity of DCs *via* the C-type lectin receptor Dectin-1.

## Data Availability Statement

The datasets generated for this study are available on request to the corresponding author.

## Ethics Statement

The studies involving human participants were reviewed and approved by Mayo Clinic and Baylor Healthcare System. The patients/participants provided their written informed consent to participate in this study. The animal study was reviewed and approved by California National Primate Research Center, University of California, Davis, CA, United States.

## Author Contributions

CG, KU, MW, and HJ conducted experiments and analyzed the results. SZ generated anti-hDectin-1 mAb. MM provided clinical samples from allergy patients. JH and RK generated the anti-hDectin-1-Pam_3_CSK_4_ conjugate. LM performed the *in vivo* experiments on non-human primates. CG, KU, and SO designed the experiments. CG and SO wrote and edited the manuscript with input from all coauthors. SO supervised this study. All authors contributed to the article and approved the submitted version.

## Funding

This work was supported by the NIAID/NIH (1 R21 AI101810-01, SO) and American Asthma Foundation (AAF15-0038, SO).

## Conflict of Interest

The authors declare that the research was conducted in the absence of any commercial or financial relationships that could be construed as a potential conflict of interest.
