# Supplementary material for: Dectin-1 Controls TSLP-Induced Th2 Response by Regulating STAT3, STAT6, and p50-RelB Activities in Dendritic Cells
Source: Front Immunol. 2021 Jul 7;12:678036. doi: 10.3389/fimmu.2021.678036 (PMC8293820; doi:10.3389/fimmu.2021.678036)
Supplement: Supplementary file 1 [file DataSheet_1.pdf]

Supplementary Material

Dectin-1 Controls TSLP-Induced Th2 Response by Regulating STAT3, STAT6, and p50-RelB Activities in Dendritic Cells

Chao Gu,[1] Katherine Upchurch,[2] Joshua Horton,[2] Mathew Wiest,[1,2] Sandra Zurawski,[3] Mark Millard,[4] Robert Kane,[2,5] HyeMee Joo,[1,2] Lisa Miller,[6] and SangKon Oh[1,2,*]

[1]Department of Immunology, Mayo Clinic, Scottsdale, AZ, United States.
[2]Institute of Biomedical Studies, Baylor University, Waco, TX, United States.
[3]Baylor Research Institute, Dallas, TX, United States.
[4]Department of Pulmonology, Baylor University Medical Center, Dallas, TX, United States.
[5]Department of Chemistry and Biochemistry, Baylor University, Waco, TX, United States.
[6]California National Primate Research Center, University of California, Davis, CA, United States.
[*]Correspondence: Oh.SangKon@mayo.edu

This file includes:

Supplementary Figure 1. Reaction scheme for preparation of anti-hDectin-1-Pam_3CSK_4 conjugate from anti-hDectin-1 mAb and Pam_3CSK_4.

Supplementary Figure 2. Curdlan decreases OX40L expression on mDCs which encounters TSLP first.

Supplementary Figure 3. TSLP induces differential OX40L expression on mDC subsets.

Supplementary Figure 4. Dectin-1 activation suppresses TSLP-induced Th2 cell priming.

Supplementary Figure 5. Curdlan-mediated decrease of Th2 response is dependent on OX40L.

Supplementary Figure 6. Curdlan-mediated suppression of TSLP-induced OX40L expression is dependent on Syk and STAT6.

Supplementary Figure 7. Expression of TSLP receptor complex on mDCs upon TSLP and/or curdlan treatment.

Supplementary Figure 8. Syk, IL-10 and TNFα are involved in nuclear p100 accumulation in mDCs upon TSLP and curdlan treatment.

Supplementary Figure 9. Syk and IL-10 are involved in regulating STAT6 activity in mDCs upon TSLP and curdlan treatment.

Supplementary Figure 10. Anti-hDectin-1-Pam_3CSK_4 conjugate decreases TSLP-induced inflammatory Th2 cell priming.

Supplementary Figure 11. Pam_3CSK_4 alone does not decrease TSLP-induced inflammatory Th2 cell priming.

Supplementary Figure 12. Pilot *in vivo* experiment in non-human primates.

Supplementary Table 1. Allergy patient information.

Supplementary Figure 1

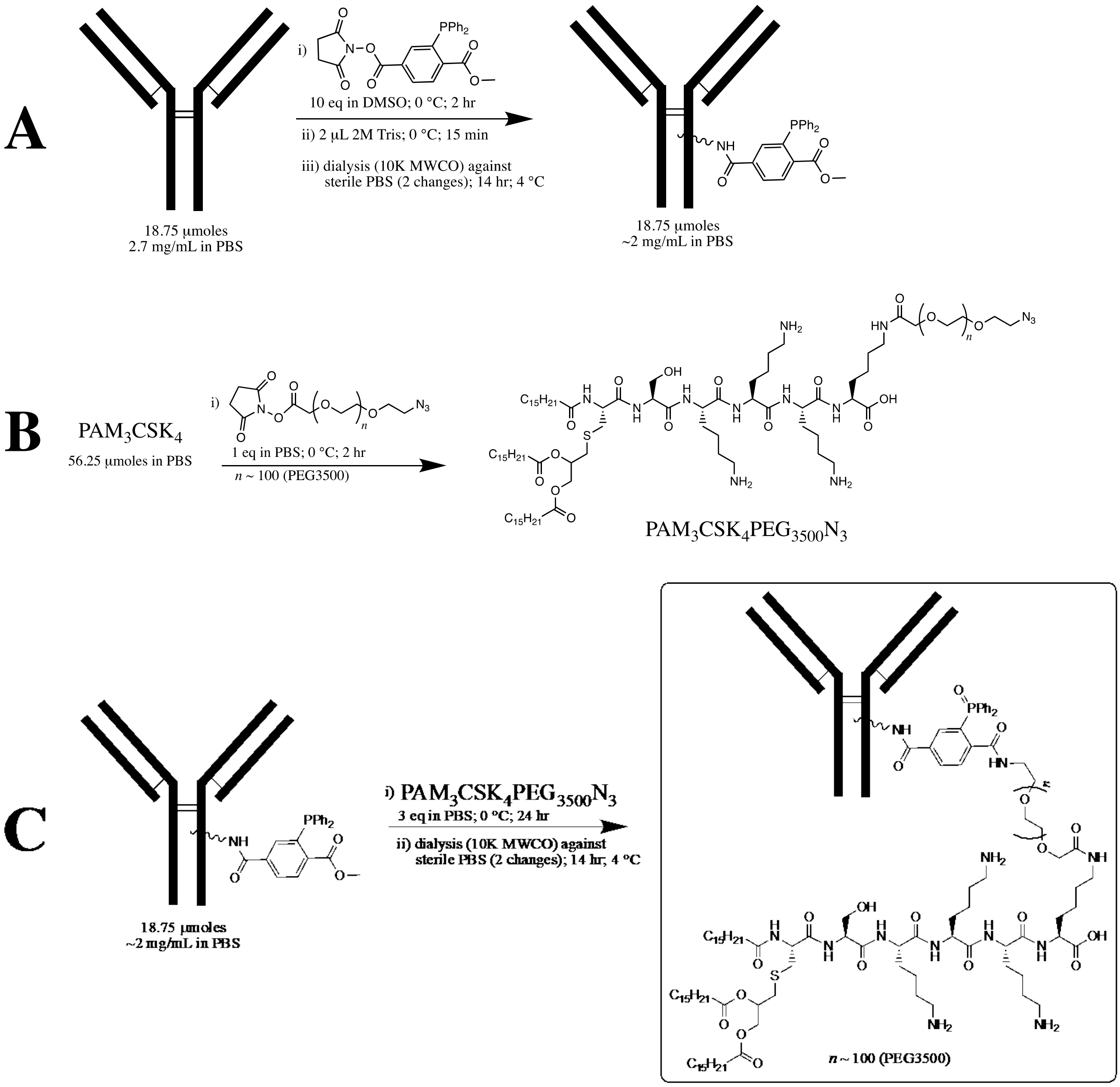

Supplementary Figure 1. Reaction scheme for preparation of anti-hDectin-1-Pam$_3$CSK$_4$ conjugate from anti-hDectin-1 mAb and Pam$_3$CSK$_4$.

Supplementary Figure 2

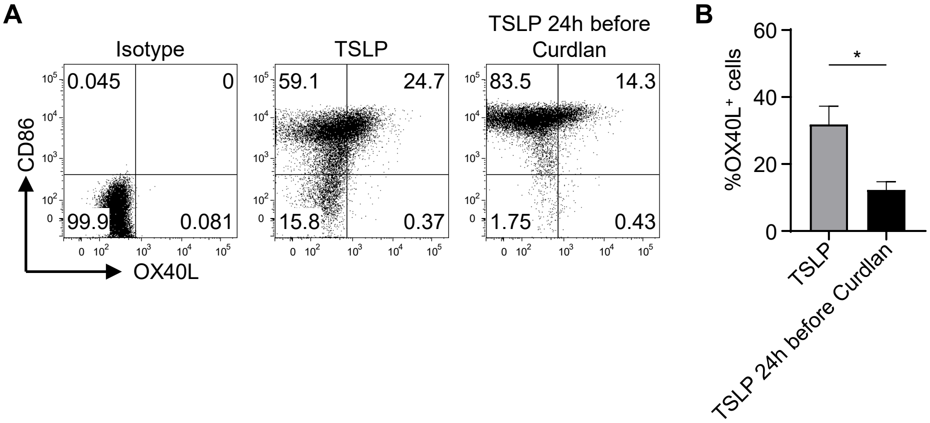

Supplementary Figure 2. Curdlan decreases OX40L expression on mDCs which encounter TSLP first.

(A, B) Sorted mDCs were incubated with TSLP alone for 48h or first treated with TSLP for 24h before incubation with curdlan for another 24h. Expression of CD86 and OX40L was then investigated by flow cytometry staining. Live singlets were gated for analysis. Gating on CD86 and OX40L was based on isotype control staining. Representative FACS data **(A)** and summarized data **(B)** from different donors (n = 3) are shown. Data in **(B)** are represented as mean ± SD. Significance was determined using paired t test (two-tailed). *$p < 0.05$ for the comparison between groups.

Supplementary Figure 3

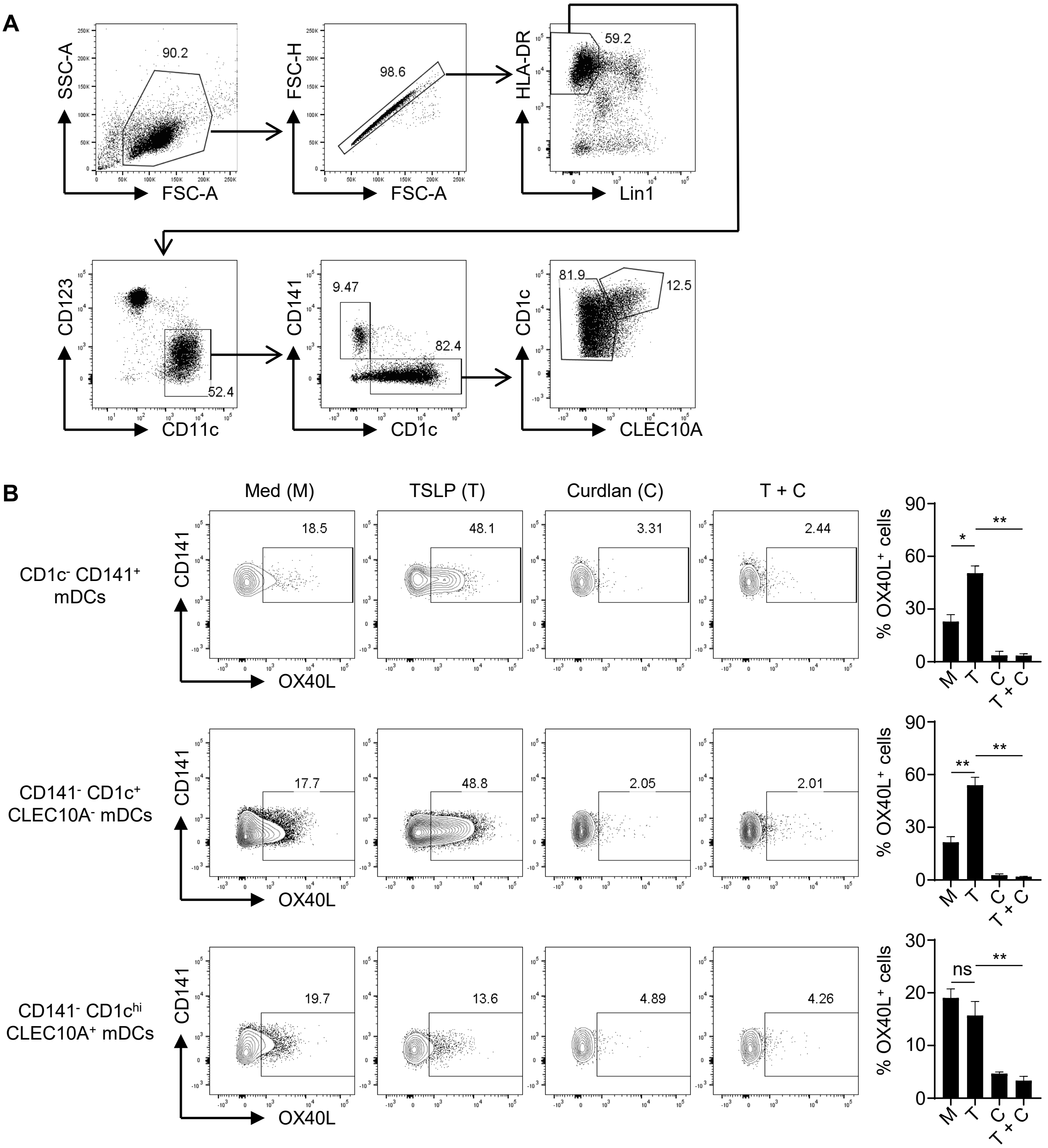

Supplementary Figure 3. TSLP induces differential OX40L expression on mDC subsets.
(A) Pan-DCs were first enriched from fresh PBMCs using enrichment kit before flow cytometry cell sorting. $Lin1^{-}DR^{+}CD11c^{+}CD123^{-}$ cells were sorted as mDCs and the frequency of three mDC subsets was analyzed by gating $CD1c^{-}CD141^{+}$ population, $CD141^{-}CD1c^{hi}CLEC10A^{+}$ population and $CD141^{-}CD1c^{+}CLEC10A^{-}$ population. Representative FACS data from three independent experiments is shown. **(B)** After 48h incubation with TSLP and/or curdlan, expression of OX40L on mDC subsets was investigated. Representative FACS data (left panel) and summarized data (right panel) of OX40L expression from different donors (n = 3) are shown. Data in **(B, right panel)** are represented as mean ± SD. Significance was determined using one-way ANOVA with Tukey multiple comparisons test for **(B)**. *$p < 0.05$, **$p < 0.01$, ***$p < 0.001$ for the comparison between groups.

Supplementary Figure 4

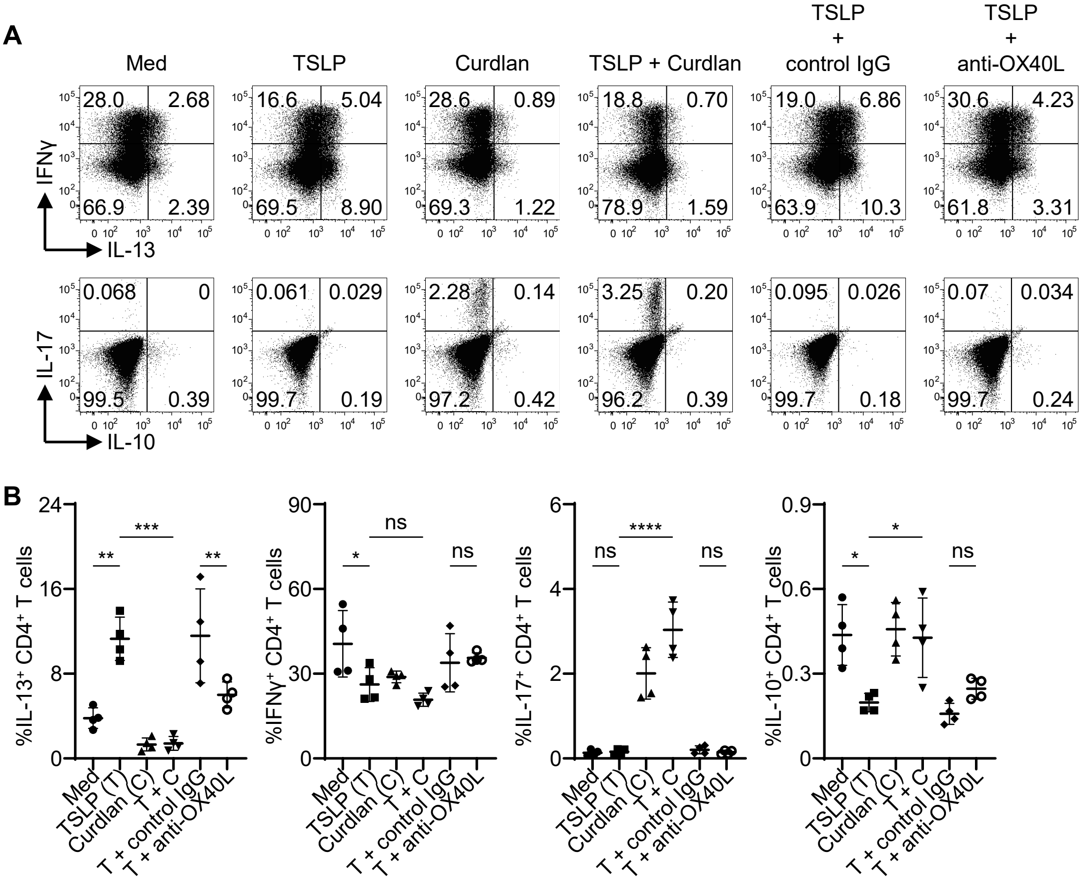

Supplementary Figure 4. Dectin-1 activation suppresses TSLP-induced Th2 cell priming.
(A, B) $CD4^+$ T cell differentiation was assessed by intracellular staining of cytokines. Sorted mDCs were incubated with medium (Med), TSLP, curdlan or both for 24h before coculture with allogenic naïve $CD4^+$ T cells for 7d. In some groups, mDCs were pre-incubated with anti-OX40L or isotype control before coculture with T cells. Live singlets were gated for analysis. Representative FACS data **(A)** and summarized data **(B)** from four independent experiments with cells from different donors are shown. Data in **(B)** are represented as mean ± SD. Significance was determined using one-way ANOVA with Tukey multiple comparisons test for **(B)**. *$p < 0.05$, **$p < 0.01$, ***$p < 0.001$, ****$p < 0.0001$ for the comparison between groups.

Supplementary Figure 5

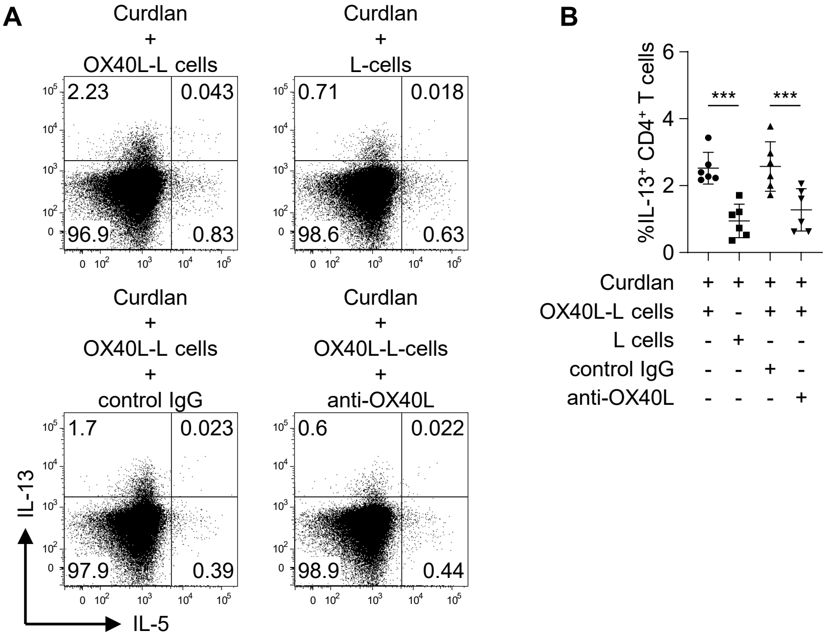

Supplementary Figure 5. Curdlan-mediated decrease of Th2 response is dependent on OX40L. **(A, B)** $CD4^+$ T cells primed by Dectin-1-activated mDCs with or without OX40L signal were investigated by intracellular staining of cytokines. Representative FACS data **(A)** and summarized data **(B)** from different donors (n = 6) are shown. Data in **(B)** are represented as mean ± SD. Significance was determined using one-way ANOVA with Tukey multiple comparisons test for **(B)**. ***$p < 0.001$ for the comparison between groups.

Supplementary Figure 6

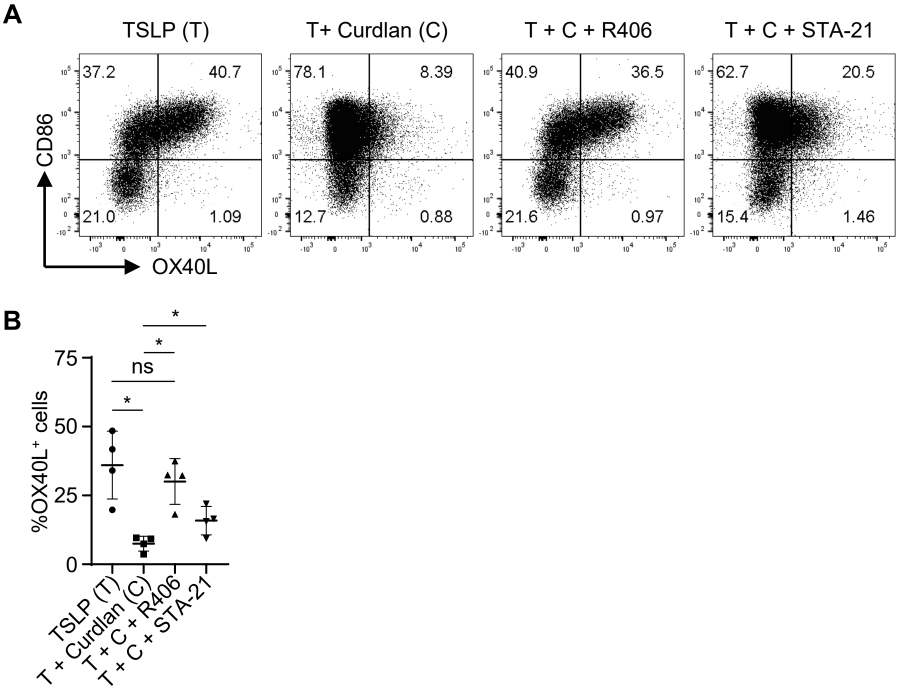

Supplementary Figure 6. Curdlan-mediated suppression of TSLP-induced OX40L expression is dependent on Syk and STAT6.

(A, B) Sorted mDCs were pre-incubated with Syk inhibitor R406 or STAT3 inhibitor STA-21 for 1h before stimulation with TSLP and/or curdlan for 48h followed by surface staining of OX40L. Live singlets were gated for analysis. Representative FACS data **(A)** and summarized data **(B)** of OX40L expression from different donors (n = 4) are shown. Data in **(B)** are represented as mean ± SD. Significance was determined using one-way ANOVA with Tukey multiple comparisons test for **(B)**. **p* < 0.05 for the comparison between groups.

Supplementary Figure 7

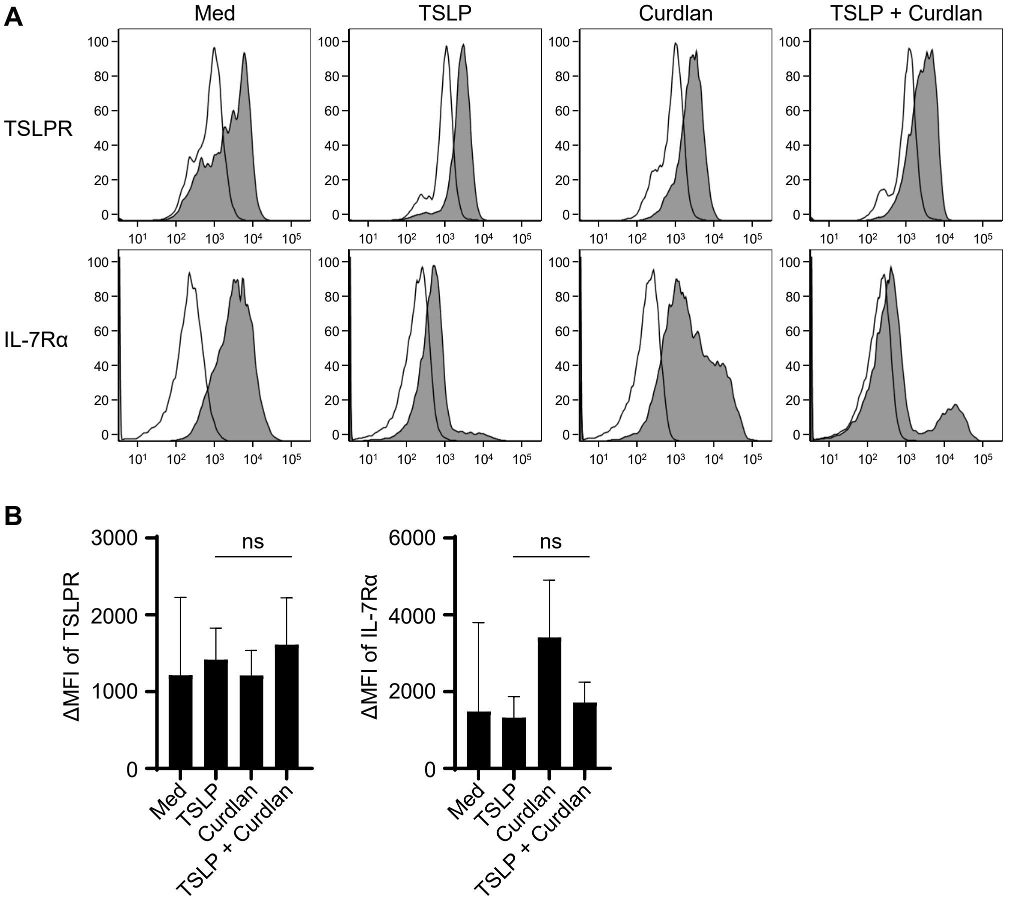

Supplementary Figure 7. Expression of TSLP receptor complex on mDCs upon TSLP and/or curdlan treatment.

(**A, B**) Sorted mDCs were treated by indicated reagents for 40h before staining of TSLPR and IL-7Rα (tinted histogram), open histogram denotes isotype control. Representative FACS data (**A**) and summarized data of mean fluorescence intensity (MFI) after isotype subtraction (**B**) generated from three experiments with different donors are shown. Significance was determined using one-way ANOVA with Tukey multiple comparisons test for **(B)**.

Supplementary Figure 8

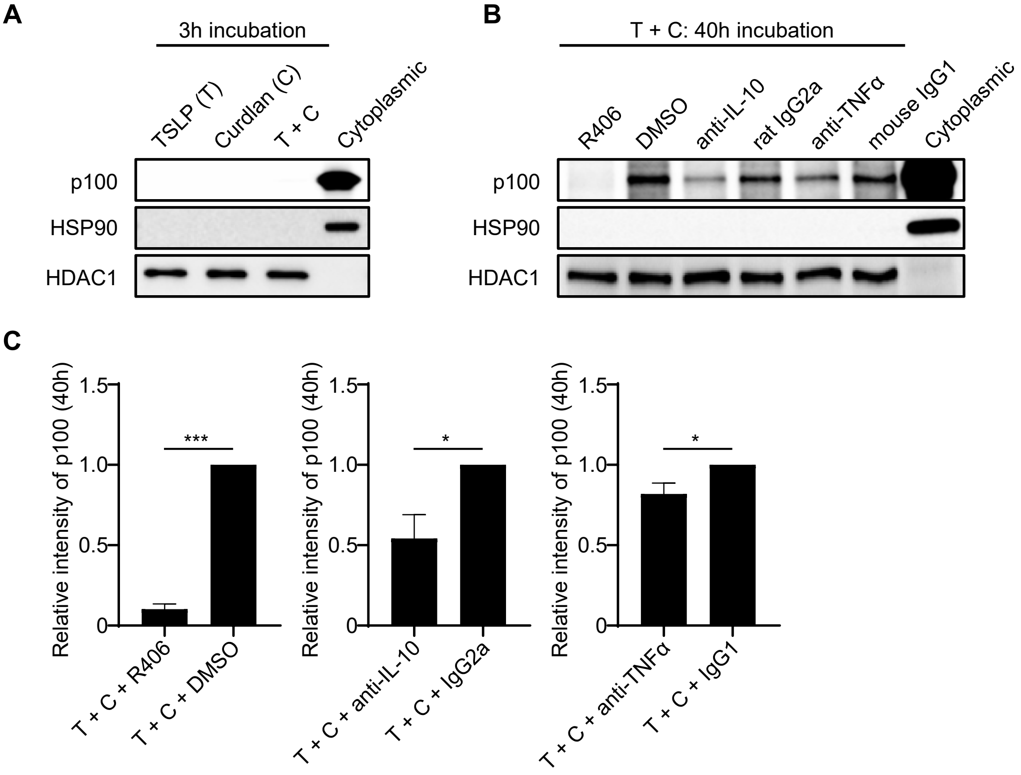

Supplementary Figure 8. Syk, IL-10 and TNFα are involved in nuclear p100 accumulation in mDCs upon TSLP and curdlan treatment.
(A) Nuclear proteins from mDCs treated with TSLP and/or curdlan for 3h were used for immunoblotting. Cytoplasmic protein served as control for detection of p100. HDAC1 was probed as loading control and HSP90 was probed to investigate the possible cytoplasmic contamination in nuclear extraction. The Western blot data shown is representative of three independent experiments. **(B, C)** Sorted mDCs were pre-incubated with Syk inhibitor R406, anti-IL-10, anti-TNFα or isotype control for 1h before stimulation with TSLP and curdlan for 40h. Nuclear proteins were used for immunoblotting. Cytoplasmic protein served as control for detection of p100. Representative western blot data **(B)** and summarized data **(C)** from three independent experiments are shown. The intensity of p100 was first normalized to that of HDAC1 in the same group, and then relative intensity was obtained by comparison with DMSO or isotype group. Data in **(C)** are represented as mean ± SD. Significance was determined using paired t test (two-tailed) for **(B)**. *$p < 0.05$, ***$p < 0.001$ for the comparison between groups.

Supplementary Figure 9

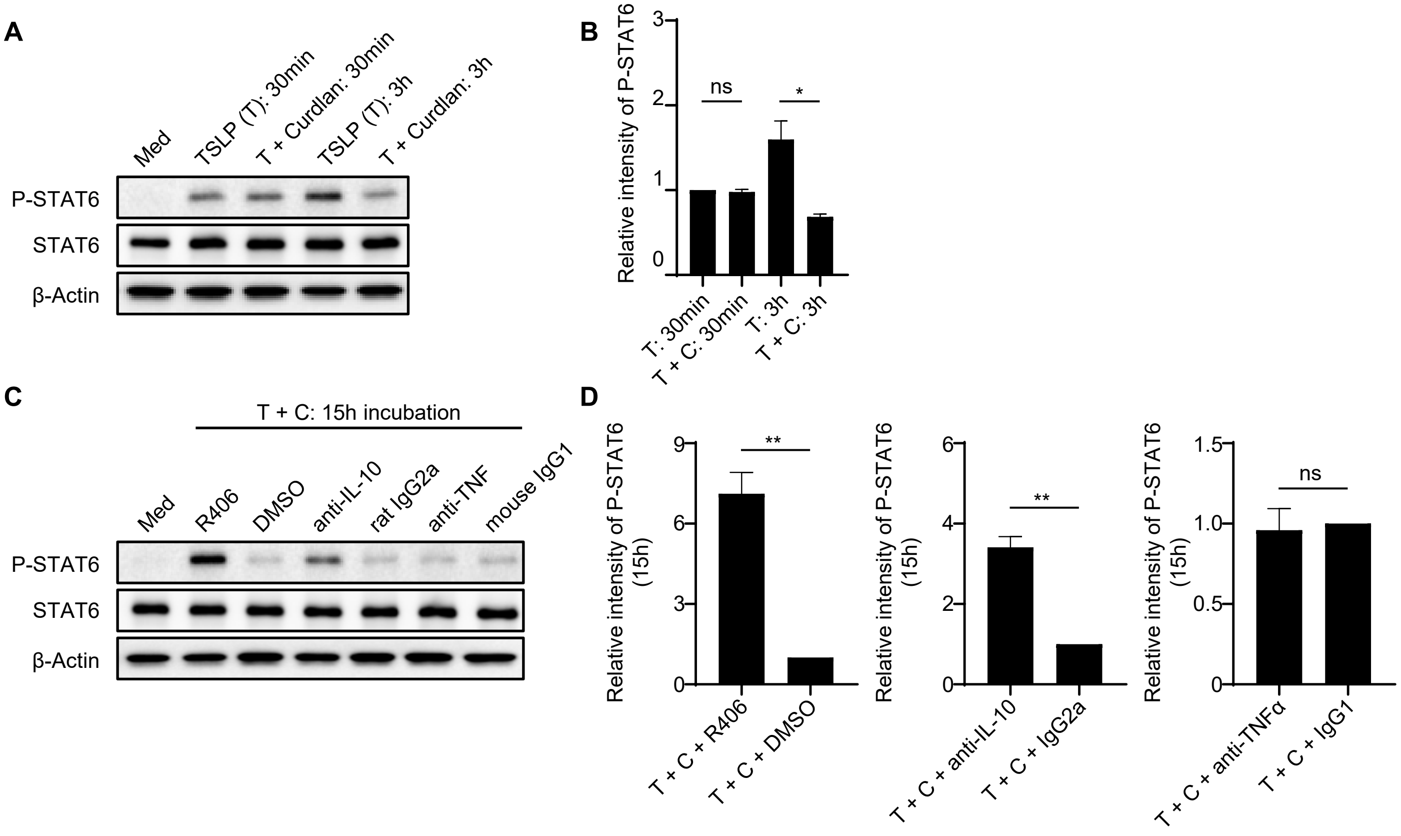

Supplementary Figure 9. Syk and IL-10 are involved in regulating STAT6 activity in mDCs upon TSLP and curdlan treatment.

(A, B) Whole cell lysates from mDCs treated with TSLP and/or curdlan for 30min or 3h were used for immunoblotting. Representative Western blot data (**A**) and summarized data (**B**) from different donors (n = 3) after 30min or 3h stimulation are shown. The intensity of P-STAT6 was first normalized to that of STAT6 in the same group, and then relative intensity was obtained by comparison with TSLP 30min stimulation group.**(C, D)** Sorted mDCs were pre-incubated with Syk inhibitor R406, anti-IL-10, anti-TNFα or isotype control for 1h before stimulation with TSLP and curdlan for 15h. Whole cell lysates were used for immunoblotting. Representative western blot data **(C)** and summarized data **(D)** from three independent experiments are shown. The intensity of P-STAT6 was first normalized to that of STAT6 in the same group, and then relative intensity was obtained by comparison with DMSO or isotype group. Data in **(B, D)** are represented as mean ± SD. Significance was determined using one-way ANOVA with Tukey multiple comparisons test for **(B)** and paired t test (two-tailed) for **(D)**. $*p < 0.05$, $**p < 0.01$ for the comparison between groups.

Supplementary Figure 10

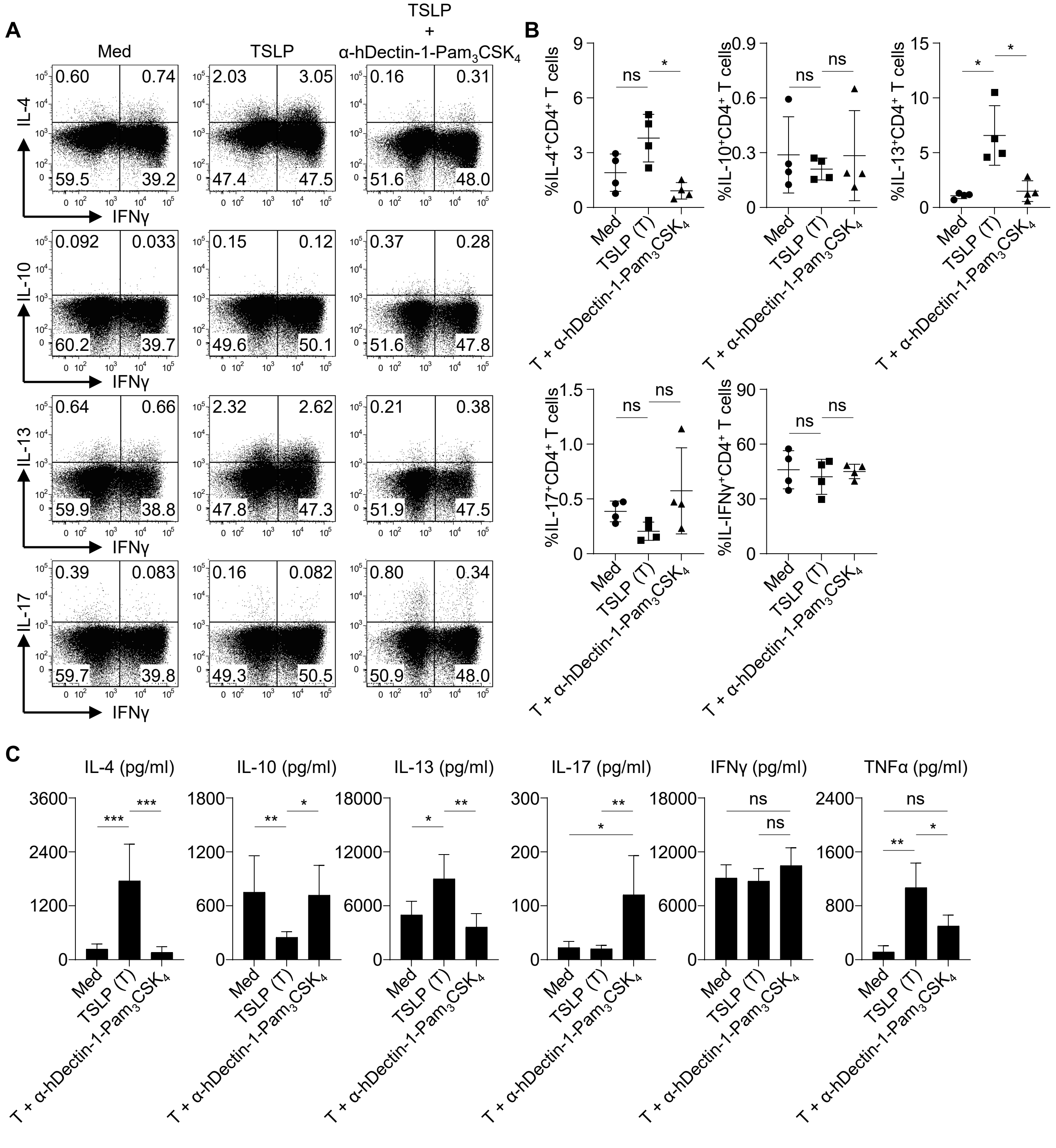

Supplementary Figure 10. Anti-hDectin-1-Pam$_3$CSK$_4$ conjugate decreases TSLP-induced inflammatory Th2 cell priming.

(A, B) Sorted mDCs were incubated with indicated reagents for 24h before coculture with allogenic naïve CD4$^+$ T cell for 7d. CD4$^+$ T cell differentiation was assessed by intracellular staining of cytokines. Live singlets were gated for analysis. Representative FACS data **(A)** and summarized data **(B)** from different donors (n = 4) are shown. **(C)** After 5d coculture of mDCs and CD4$^+$ T cells as in **(A)**, T cells were stimulated by anti-CD3/28 beads for 48h before measuring cytokine production in culture supernatants by multiplex assay. Summarized data from different donors (n = 6) are shown. Data in **(B, C)** are represented as mean ± SD. Significance was determined using one-way ANOVA with Tukey multiple comparisons test for **(B, C)**. *p < 0.05, **p < 0.01, ***p < 0.001 for the comparison between groups.

Supplementary Figure 11

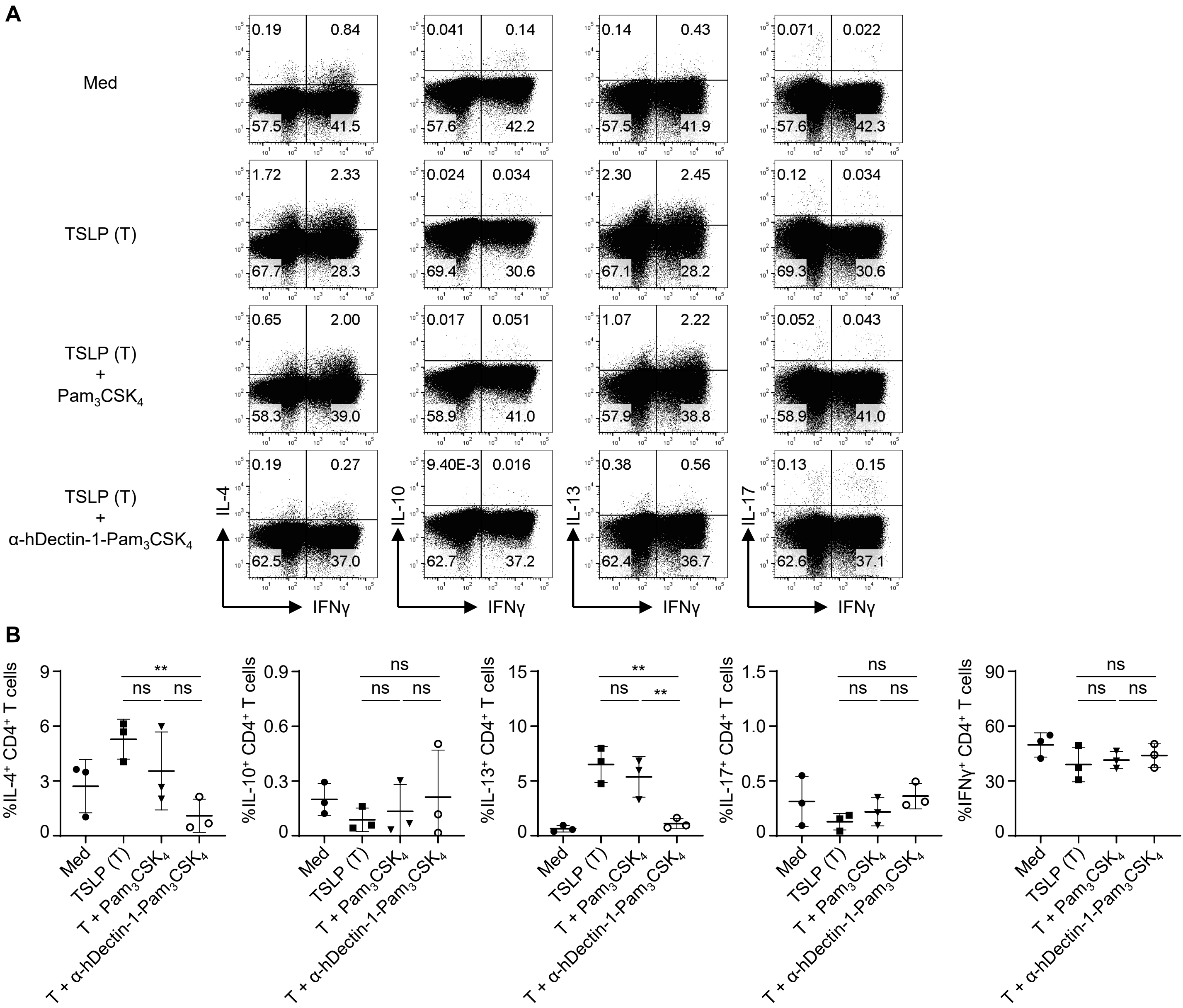

Supplementary Figure 11. Pam$_3$CSK$_4$ alone does not decrease TSLP-induced inflammatory Th2 cell priming. (A, B) Sorted mDCs were incubated with indicated reagents for 24h before coculture with allogenic naïve CD4$^+$ T cell for 7d. CD4$^+$ T cell differentiation was assessed by intracellular staining of cytokines. Live singlets were gated for analysis. Representative FACS data **(A)** and summarized data **(B)** from different donors (n = 3) are shown. Data in **(B)** are represented as mean ± SD. Significance was determined using one-way ANOVA with Tukey multiple comparisons test for **(B)**. *$p < 0.05$, **$p < 0.01$ for the comparison between groups.

Supplementary Figure 12

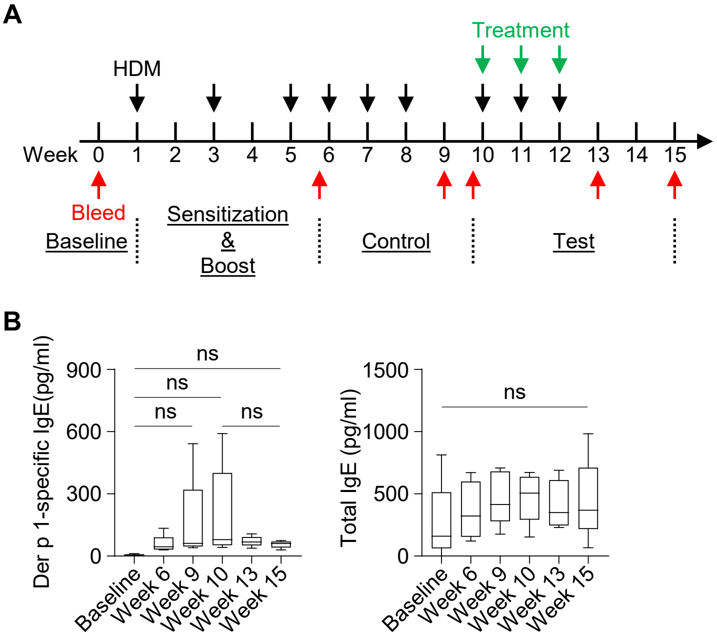

Supplementary Figure 12. Pilot *in vivo* experiment in non-human primates.
(A) Scheme of the first in vivo test in NHP. Injection of HDM extract, bleeding, and treatment with anti-hDectin-1-Pam_3CSK_4 conjugate was performed as indicated. **(B)** Der p 1-specific IgE and total IgE in the serum of animals at indicated time points were measured by ELISA. Data in **(B)** are represented as box and whiskers (min to max). Significance was determined using one-way ANOVA with Tukey multiple comparisons test for **(B)**. ns, not significant.

Supplementary Table 1

Table S1. Allergy patient information

Patient #	Sex	Age	Ethnicity	Symbol
1	F	33	AA	●
2	F	57	C	■
3	M	52	AA	▲
4	M	49	NA	▼
5	F	42	AA	◆
6	F	51	C	○
7	M	50	C	□
8	F	56	C	△

F: female. M: male.
AA: African American. C: Caucasian. NA: Native American.
All patients showed positive response to ragweed in skin prick test.
